# Exogenous auxin regulates multi-metabolic network and embryo development, controlling seed secondary dormancy and germination in *Nicotiana tabacum* L.

**DOI:** 10.1186/s12870-016-0724-5

**Published:** 2016-02-09

**Authors:** Zhenhua Li, Jie Zhang, Yiling Liu, Jiehong Zhao, Junjie Fu, Xueliang Ren, Guoying Wang, Jianhua Wang

**Affiliations:** College of Agriculture and Biotechnology, China Agricultural University, Yuanmingyuan West Road, Beijing, 100094 China; Molecular Genetics Key Laboratory of China Tobacco, Guizhou Academy of Tobacco Science, GuiYang, 550081 China; Institute of Tobacco, Guizhou University, Guiyang, 550025 China; Institute of Crop Sciences, Chinese Academy of Agricultural Sciences, Beijing, 100081 China

**Keywords:** Auxin, Seed dormancy, Germination, Wateruptake, Embryo development, Endosperm burst, Hormone, *Nicotiana tabacum*, RNA-seq

## Abstract

**Background:**

Auxin was recognized as a secondary dormancy phytohormone, controlling seed dormancy and germination. However, the exogenous auxin-controlled seed dormancy and germination remain unclear in physiological process and gene network.

**Results:**

Tobacco seeds soaked in 1000 mg/l auxin solution showed markedly decreased germination compared with that in low concentration of auxin solutions and ddH_2_O. Using an electron microscope, observations were made on the seeds which did not unfold properly in comparison to those submerged in ddH_2_O. The radicle traits measured by WinRHIZO, were found to be also weaker than the other treatment groups. Quantified by ELISA, there was no significant difference found in β-1,3glucanase activity and abscisic acid (ABA) content between the seeds imbibed in gradient concentration of auxin solution and those soaked in ddH_2_O. However, gibberellic acid (GA) and auxin contents were significantly higher at the time of exogenous auxin imbibition and were gradually reduced at germination. RNA sequencing (RNA-seq), revealed that the transcriptome of auxin-responsive dormancy seeds were more similar to that of the imbibed seeds when compared with primary dormancy seeds by principal component analysis. The results of gene differential expression analysis revealed that auxin-controlled seed secondary dormancy was associated with flavonol biosynthetic process, gibberellin metabolic process, adenylyl-sulfate reductase activity, thioredoxin activity, glutamate synthase (NADH) activity and chromatin regulation. In addition, auxin-responsive germination responded to ABA, auxin, jasmonic acid (JA) and salicylic acid (SA) mediated signaling pathway (red, far red and blue light), glutathione and methionine (Met) metabolism.

**Conclusions:**

In this study, exogenous auxin-mediated seed secondary dormancy is an environmental model that prevents seed germination in an unfavorable condition. Seeds of which could not imbibe normally, and radicles of which also could not develop normally and emerge. To complete the germination, seeds of which would stimulate more GA synthesis to antagonize the stimulation of exogenous auxin. Exogenous auxin regulates multi-metabolic networks controlling seed secondary dormancy and germination, of which the most important thing was that we found the auxin-responsive seed secondary dormancy refers to epigenetic regulation and germination to enhance Met pathway. Therefore, this study uncovers a previously unrecognized transcriptional regulatory networks and physiological development process of seed dormancy and germination with superfluous auxin signal activate.

**Electronic supplementary material:**

The online version of this article (doi:10.1186/s12870-016-0724-5) contains supplementary material, which is available to authorized users.

## Background

The transition from dormancy to germination in seeds is a key physiological process during the life-cycle of some plants. Water uptake, seed dormancy released, embryo expansion, and radical breakthrough of seed envelopes is considered as the completion of the seed germination [1]. Plant hormone, as a signaler, is also important for seed dormancy and germination [2]. GA and ABA are recognized as the key internal factors, with GA promoting and ABA inhibiting seed germination and dormancy [3]. Recent studies support the fact that the ABA/GA ratio regulates the metabolic transition required for dormancy release and germination [4–6]. On this basis, it is inferred that other hormones such as ethylene [7, 8] and cytokinin [9, 10] also influence germination through cross-talk mechanisms.

Auxin, as a versatile trigger taking part in many plant developmental processes [11], also plays a critical role in root development, such as shaping the embryonic root pole, determining the root meristem size and controlling root cell elongation [12]. However, the biological function of auxin in seed dormancy and germination is yet to be explored. Recently, a previously unrecognized regulatory factor of seed dormancy, auxin, was identified as a secondary dormancy hormone. It works with ABA signaling to control seed dormancy and germination [13].

Numerous genes involved in dormancy and germination regulation have already been confirmed. ABA receptors (*PYR, PYL/RCAR*), protein phosphatase 2C (*ABI1, ABI2, HAB1, AHG3*), protein kinase (*SnRK2.2, 2.3, 2.6*) and other dormancy hormone regulators were reviewed recently [14]. PIL5/PIF1 has been identified as an important upstream component, which through transcriptional control of biosynthetic genes, reciprocally regulates levels of both ABA and GA [12], and PIL5 was found to target promoters of various hormonal signaling genes including *ARF18, IAA16, CRF2,* and *JAZ1* [15]. Very recently, it was reported that auxin acts upstream of the major regulator of seed dormancy, by recruiting the auxin response factors ARF 10 and 16 to control the expression of ABI3 during seed germination [13].

However, the mechanisms for the auxin regulation of seed germination process and the genetic response to exogenous auxin stimulation on a transcriptomic scale remained unknown. The aim of this study is to analyze the differential physiological process and genes expression of the auxin-controlled seed dormancy and germination using *Nicotiana tabacum* L as the model plant. This is the first study that exogenous auxin-controlled seed dormancy and germination on a transcriptomic scale and physiological development process also would be taken into consideration.

## Results

### Seed dormancy depends on exogenous auxin levels

**Fig. 1:**
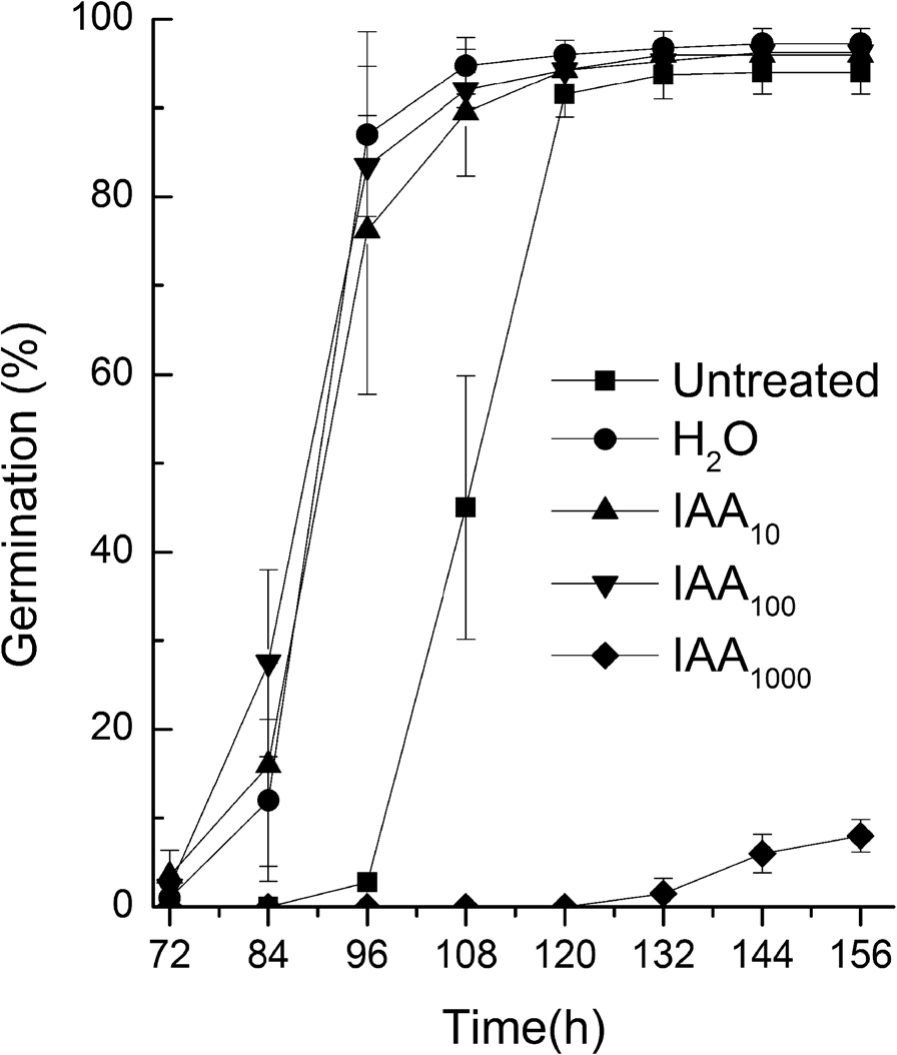
A graphical representation of exogenous auxin levels controlling seed dormancy. Germination of seeds imbibed in supplementation IAA solution or ddH_2_O 24 h or untreated and then germinated on filter paper beds 156 h. Subscript 10, 100, 1000 indicated the concentration gradients

Keeping in mind auxin control of seed dormancy in *Arabidopsis* [13], it was presumed that the exogenous high concentration of auxin might promote seed secondary dormancy in tobacco. We found that tobacco seeds soaked in 1000 mg/l indole-3-acetic acid (IAA) solution showed markedly decreased germination compared with those soaked in 0, 10 and 100 mg/l IAA solution and even unsoaked seeds (Fig. 1).

### Exogenous auxin regulates the emergence of germinated seed radicle

**Fig. 2:**
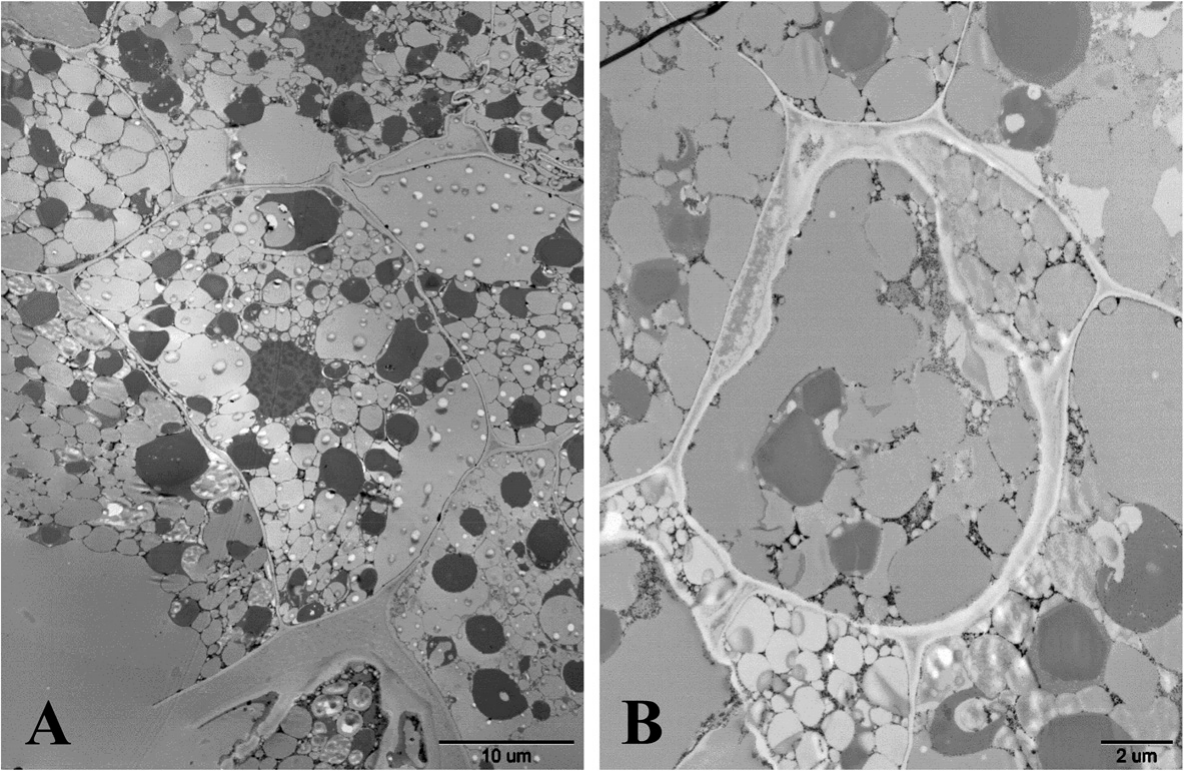
An electron micrograph showing (**a**) seed secondary dormancy, vacuole of the seeds not fully expanded, and many follicles bubble were found in cells of the seeds that treated with IAA (1000 mg · L^−1^) 24 h, (**b**) seed germinating, vacuole of seeds treated with ddH_2_O 24 h and vacuole of which completely expanded

**Fig. 3:**
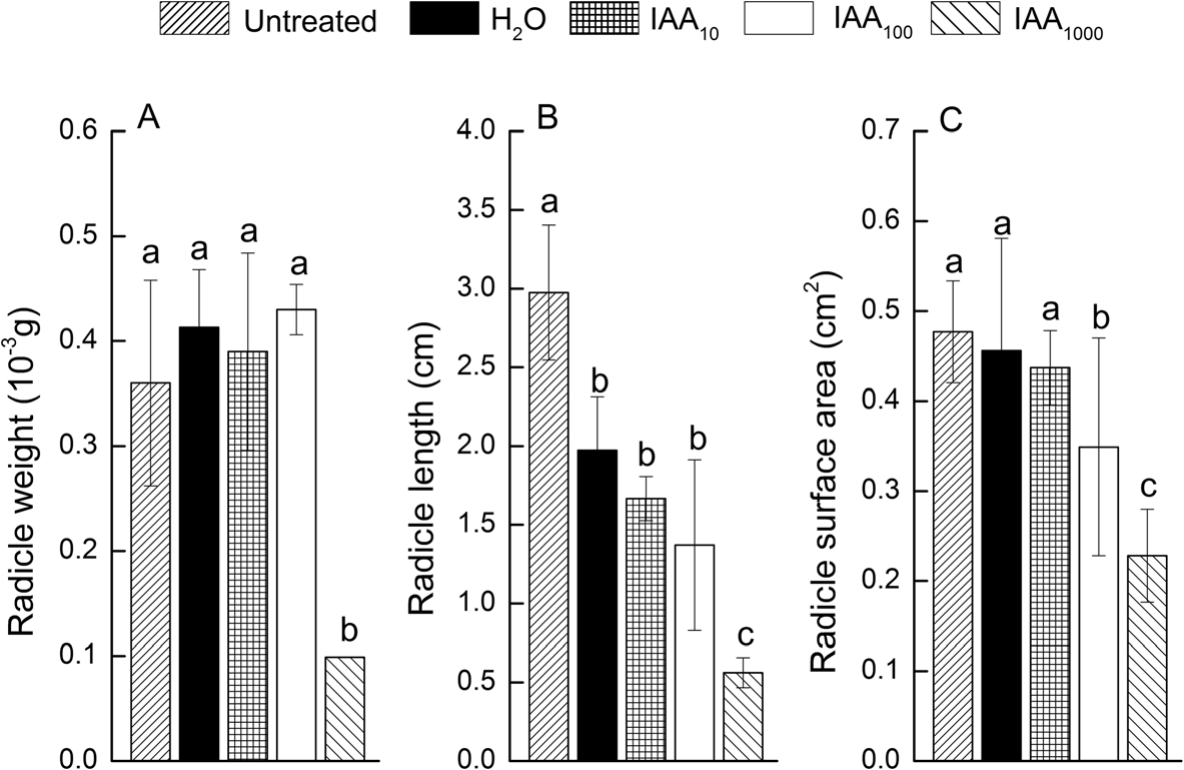
Radicle weight (**a**), length (**b**) and surface area (**c**) of tobacco seed imbibed in supplementation IAA solution or ddH_2_O 24 h or untreated and then germinated on filter paper beds 156 h. Different letters indicate significant differences according to Duncan test (means ± SD, p < 0.05). Subscript 10, 100, 1000 indicate the concentration gradients

**Fig. 4:**
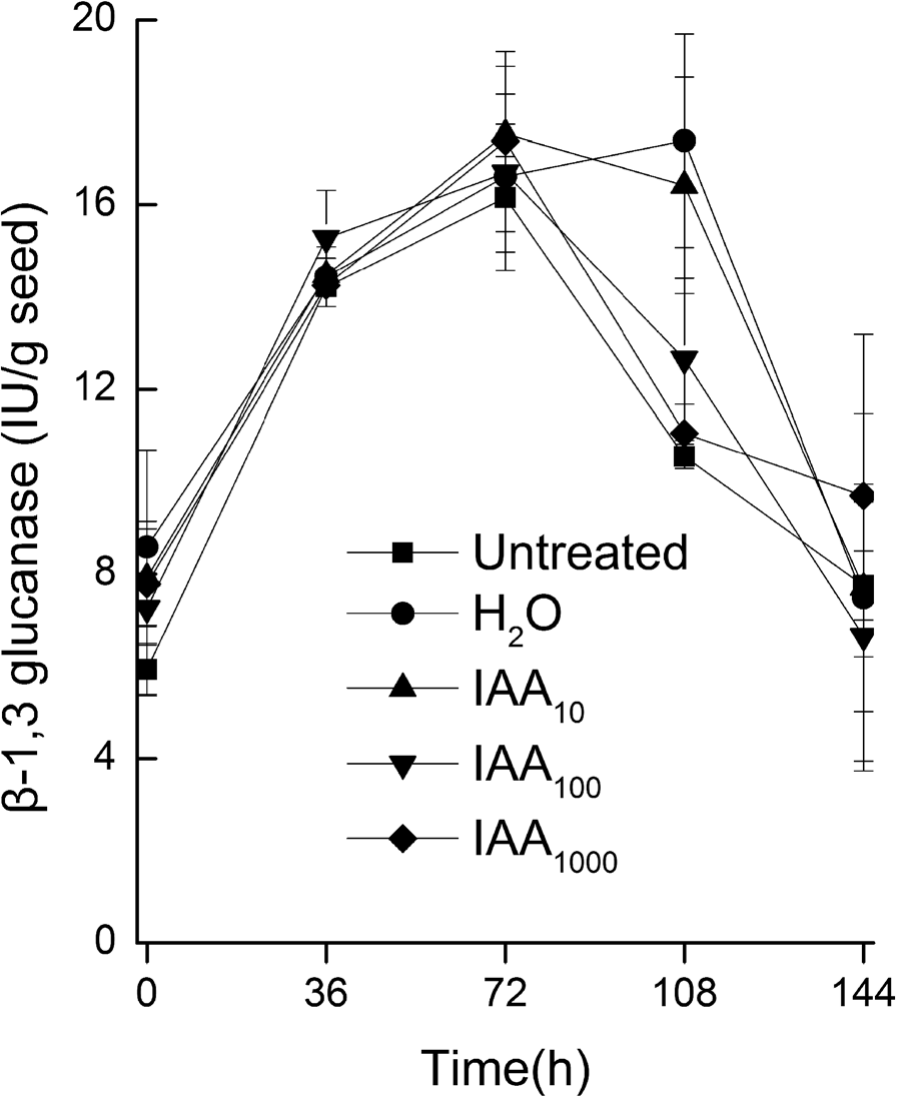
β-1, 3glucanase activity (means ± SD) of tobacco seed imbibed in supplementation IAA solution or ddH_2_O 24 h or untreated and then germinated on filter paper beds 144 h. Subscript 10, 100, 1000 indicate the concentration gradients

The effect of exogenous auxin on seed germination including water uptake, radicle emergence, endosperm burst and cotyledons unfolding assay were analyzed. The result showed that vacuoles of seeds imbibed in 1000 mg/l IAA solution could not properly unfold compared with that in ddH_2_O (Fig. 2a and b). Also the radicle traits, which included the radicle weight, length and surface area, were significantly weaker (Fig. 3a-c), but there was no significant difference in β-1,3glucanase activity (Fig. 4). The radicle of all the seeds did not emerge even when approximately half of the cotyledons unfolded in the germination process.

### Gibberellin level was regulated by exogenous auxin and not abscisic acid

**Fig. 5:**
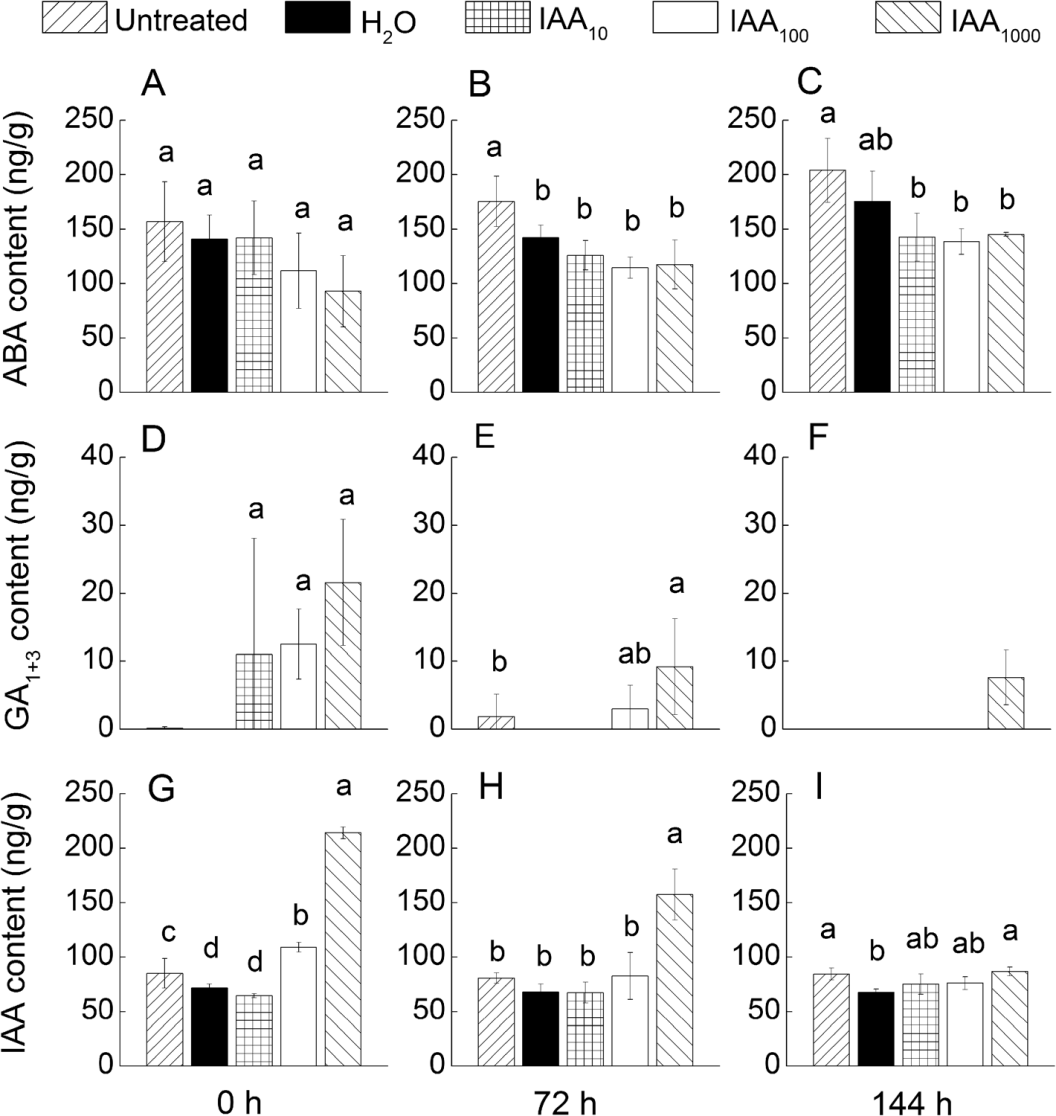
Endogenous ABA (**a**-**c**), GA1+3 (**d**-**f**) and IAA (**g**-**i**) hormones’ content of tobacco seed imbibed in supplementation IAA solution or ddH_2_O 24 h or untreated and then germinated on filter paper beds 144 h. Subscript 10, 100, 1000 indicate the concentration gradients

The effect of exogenous IAA on content of endogenous hormones including ABA, GA, and IAA were examined. The dynamic changes in ABA, GA_1+3_ and IAA in three germination stages were shown in Fig. 5. The result indicated that there was no significant difference in ABA content between seeds imbibed in gradient concentration of auxin solution and those soaked in ddH_2_O, in all three germination stages (Fig. 5a-c). However, the GA_1+3_ (Fig. 5d-f) and IAA (Fig. 5g-i) contents were significantly higher, especially in the first stage.

### Transcriptome analysis of exogenous auxin controlled seed dormancy and germination

**Fig. 6:**
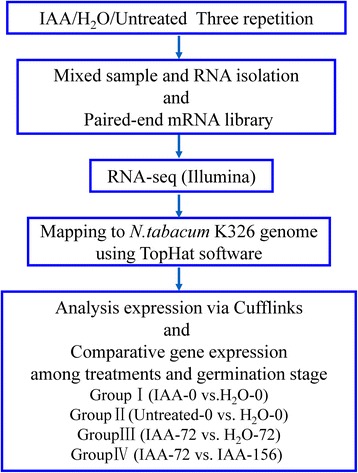
Flow chart of the RNA-seq experimental procedure

Figure 1 shows the phenotype of 72 and 156 h germinating tobacco seeds and Fig. 6 illustrates the process flux of the RNA-seq experiment. As shown, three replications of 0 or 1000 mg/l IAA imbibed seeds that germinated after 0, 72 or 156 h (for seeds status see Table 1) were respectively collected, mixed and then used for total RNA isolation. Afterwards, paired-end libraries were prepared and sequenced as described in the ‘Methods’. The quality of the data and the generated sequences were checked using the Fast QC software and Phred measure Score, respectively. The percentage of high-quality fragments was >80 % in all cases (20 units or more in Phred values which correspond to a sequencing error rate of 1 %). The mapped data generated was shown in Table 1. There were seven samples in total. An average of about 37 million high-quality paired-end reads (2 × 100 bp) for all RNA-seq samples were generated. We got approximately 263 million reads pairs, which are more than 52.6 billion bases. For each RNA-seq sample, 72.61–91.43 % (Table 1) of reads were mapped to the genome of *N.tabacum*K326 reference genome [16] with TopHat software [17]. Following this, we used the Cufflinks program [18] to reassemble the mapped reads into a set of transcripts for each sample. Then cuffmerge module in cufflinks was used to merge the transcripts from each sample to generate a unique transcript set, also named as unigenes. After removing the transcripts with length < 200 bp, a total of 107,403 unigenes were detected. These unigenes had an average length of 1757 bp and N50 value of 2105 bp, of which the lengths ranged from 200 bp to 14,848 bp. There were 80,494 (74.94 %) of the unigenes with length ≥ 1000 bp.

**Table 1:** Number of reads sequenced and mapped with Tophat. The number of unique mapping reads plus multimapping reads equals the total number of alignments. CK, H_2_O and IAA stand for imbibed tobacco seeds and control respectively; Numbers 0, 72 and 156 stand for germinated time

Sample	Seed category	Total reads	Total mapped reads	Unique mapping reads	Reads mapped in proper pairs
Untreated-0	Dormancy	39,344,236	28,566,421(72.61 %)	24,229,292(61.58 %)	25,845,450
H_2_O-0	Imbibition	39,795,012	35,183,360(88.41 %)	21,537,407(66.58 %)	29,258,962
IAA-0	Dormancy	48,297,176	38,392,739(79.49 %)	32,439,112(67.17 %)	34,779,672
Untreated-72	Germination	33,792,314	30,897,092(91.43 %)	26,584,380(78.67 %)	24,978,136
H_2_O-72	Germination	35,593,768	31,962,893(89.80 %)	27,456,500(77.14 %)	26,621,694
IAA-72	Dormancy	32,459,006	28,789,591(88.70 %)	25,040,225(77.14 %)	23,209,710
IAA-156	Germination	33,769,804	29,877,097(88.47 %)	25,821,822(76.46 %)	24,613,498

For unigenes’ functional annotation, we utilized a blastx search against the NCBI NR database, with an E-value of 1E-05 as a cut-off. The blastx search result showed that about 91,840 (85.18 %) unigenes which had significant hits in the NR database was utilized. The distribution of the unigenes best blastx hit E-value is shown in Additional file 1: Figure S1. Most of the unigenes had high similarity with NR database sequences. They include 70,439 (65.58 %) unigenes with E-value ≤ 1.0E-100, and 91,839 (85.51 %) ungenes with E-value ≤ 1.0E-5. The blastx best-hit of the 91,839 unigenes showed that the first two species with the highest hits were *Solanum lycopericum* (38,302, 41.71 %) and *Solanum tuberosum* (37,890, 41.26 %), which were the most important model organisms in Solanaceae. The distribution of best hits species is shown in Additional file 2: Figure S2. For unigenes functional classification, a blastx search against the EuKaryotic Orthologous Groups (KOG) database was done, and it showed that 60,497 unigenes matched with 25 KOG clusters. As shown in Additional file 3: Figure S3, KOG classification showed that the largest category was ‘general function prediction only’ which was same as other studies; the following category was ‘posttranslational modification, protein turnover, chaperones’. For gene ontology (GO) annotation, Blast2GO suite was used to retrieve GO terms based on the blastx search result of NCBI NR database. Among the 91,839 annotated unigenes, there were 76,969 unigenes that were annotated with at least one GO term. As a result, 62,074 unigenes were grouped into Biological Process (BP), 61,316 into Molecular Function (MF) group and 56,760 into Cellular Component (CC) group. The unigene percentage of each GO term in the three groups is shown in Additional file 4: Figure S4. Finally, KAAS tool was used to identify pathways for the unigenes. There were 23,261 (21.66 %) unigenes assigned to 2100 kyoto encyclopedia of genes and genomes (KEGG) orthologs, which were classified into 327 KEGG pathways.

**Fig. 7:**
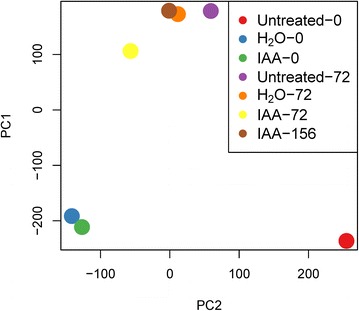
A principal component analysis (PCA) chart to explore the relationship between samples using the unigene expression quantity. The more similar the samples, the spatial distribution of different colors points more concentrated

To quantify the unigenes expression in each RNA-seq sample, we used bowtie to map the clean RNA-seq reads to all unigenes sequences, and then used eXpress software to calculate fragments per kilobase of exon per million fragments mapped (FPKM) for unigenes in each sample. To analyze the similarity of gene express patterns among these different RNA-seq samples, we firstly filtered out the unigenes without any reads mapped in all samples, and then used principal component analysis (PCA) to analyze the unigenes FPKM values of all the 7 samples. PCA plot of principal components 1 and 2 showed that the spatial distribution of H_2_O-0 and IAA-0, H_2_O-72 and IAA-156 points was more concentrated, suggesting that these samples were more similar (Fig. 7).

### Auxin-responsive dormancy seeds and primary dormancy seeds differ in their gene expression profiles

The differential expression analysis was performed by comparing untreated seeds (Primary dormancy) and auxin-imbibed seeds (Secondary dormancy) with H_2_O-imbibed seeds (No dormancy), separately. At a significant level of *p* < 0.05 and fold change ≥ 2, we identified 1958 up- and 2917 down-regulated unigenes between untreated seeds and H_2_O-imbibed seeds (Additional file 5: Figure S5A). At the same condition, we also identified 2506 up- and 2634 down-regulated unigenes between auxin-imbibed seeds and H_2_O-imbibed seeds (Additional file 5: Figure S5B). To determine the unigenes with differential expression pattern between untreated seeds and auxin-imbibed seeds relative to H_2_O-imbibed seeds, the H_2_O-imbibed seeds were taken as a control and then the unigene counts of the nine expression patterns were calculated.

**Fig. 8:**
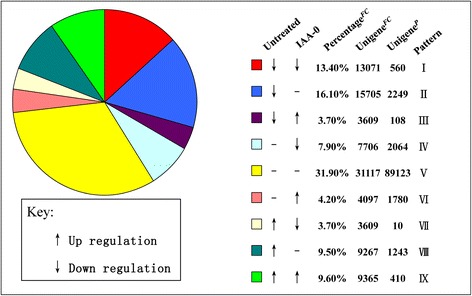
A pie chart distribution of unigenes whose expression is altered during seed dormancy. The pie chart was drawn with the percentage of fold change values. Unigenes whose expression level was significantly up-regulated or down-regulated by more than 2-fold or *p-value* < 0.05 during dormancy were grouped according to their expression behavior in the two dormancy states of untreated seeds (primary dormancy) and auxin-imbibed seeds (secondary dormancy) relative to H_2_O-imbibed seeds (non-dormancy). Arrows facing up or down represent up-regulated or down-regulated genes, respectively

As shown in Fig. 8, the *Percentage*^*FC*^ and *Unigene*^*FC*^ columns were calculated based on the unigene expression fold change, in which unigene with fold change < 0.5 was treated as down -regulated, and fold change > 2.0 was treated as up-regulated; the rest were treated as unchanged. The pie chart was drawn with the *Percentage*^*FC*^ values. The *Unigene*^*P*^ column was calculated based on the *p-values* of gene differential expression analysis, in which unigene with *p-value* < 0.05 and fold change < 0.5 was treated as down-regulated, and that with *p-value* < 0.05 and fold change > 2.0 was treated as up-regulated; the rest were treated as unchanged. In the nine expression patterns, we treated the III/IV/VI/VII patterns as the differential expression pattern between untreated seeds and auxin-imbibed seeds relative to H_2_O-imbibed seeds. The III/VII pattern had complete differential regulating tendency relative to H_2_O-imbibed seeds. In the IV/VI pattern, unigenes presented differential expression between auxin-imbibed seeds and H_2_O-imbibed seeds, but remained unchanged between untreated seeds and H_2_O-imbibed seeds. Consequently, unigenes in these four expression patterns which could also be considered as the major causal factors that led to the difference in mechanism between primary dormancy and auxin-induced secondary dormancy, and this could be used to do functional analysis.

**Table 2:** Representative significant enriched GO terms of the differential expressed unigenes

Seed status	Category	GO term	Refence set	Query set	*P-value* (FDR)	Representative gene ID	Encoded protein
Dormancy	BP	Para-aminobenzoic acid metabolic process	130	19	2.20E-04	TCONS_00105446	glucosyltransferase
		Oxalate catabolic process	6	4	2.99E-03	TCONS_00017169	4-coumarate--CoA ligase-like 10-like
		N-acylethanolamine metabolic process	34	9	6.41E-04	TCONS_00047595	fatty acid amide hydrolase-like
		Glutamate biosynthetic process	25	7	2.99E-03	TCONS_00068711	glutamate synthase 1
		Gibberellin metabolic process	127	13	3.21E-02	TCONS_00065700	gibberellin 2-oxidase 2,3,5
		Flavonol biosynthetic process	109	13	1.15E-02	TCONS_00051510	immediate-early salicylate-induced glucosyltransferase
		Cutin biosynthetic process	47	9	3.95E-03	TCONS_00032450	CYP77A19
		Auxin mediated signaling pathway	548	43	8.41E-04	TCONS_00056392,TCONS_00049526,TCONS_00102987,TCONS_00043746,TCONS_00029963,TCONS_00082867	NTGP3, Nt-iaa4.5 deduced protein, IAA9, ARF1, IAA13, LAX2
	MF	Adenylyl-sulfate reductase activity	5	4	1.43E-03	TCONS_00030231	APS reductase
		Carbon-nitrogen ligase activity, with glutamine as amido-N-donor	200	19	1.85E-02	TCONS_00077280	amidase-like protein
		Glutamate synthase (NADH) activity	22	8	3.01E-04	TCONS_00068708	glutamate synthase 1
		Indole-3-acetic acid amido synthetase activity	42	8	1.43E-02	TCONS_00011340	Nt-gh3 deduced protein
		Isoflavone 2′-hydroxylase activity	21	5	4.94E-02	TCONS_00066756	cytochrome P450
		Oxalate-CoA ligase activity	6	4	3.69E-03	TCONS_00017169	4-coumarate--CoA ligase-like 10-like
		Phosphoadenylyl-sulfate reductase (thioredoxin) activity	5	4	1.43E-03	TCONS_00030231	APS reductase
Germiantion	BP	ABA mediated signaling pathway	547	35	3.19E-03	TCONS_00022716, TCONS_00055623, TCONS_00066091	bZIP, LEB5, calcium-dependent protein kinase 8
		Auxin mediated signaling pathway	548	34	5.43E-03	TCONS_00033126, TCONS_00075562, TCONS_00084884	auxin efflux facilitator PIN3b, Nt-iaa28 deduced protein, germin like protein
		Cinnamic acid biosynthetic process	16	7	2.95E-05	TCONS_00069919	phenylalanine ammonia-lyase 4
		Cysteine biosynthetic process	547	34	5.31E-03	TCONS_00016976, TCONS_00018745, TCONS_00017791	serine acetyltransferase 7, chloroplast pigment-binding protein CP24, ZIP
		Ethylene metabolic process	247	20	4.37E-03	TCONS_00086037, TCONS_00008535, TCONS_00106286	ethylene forming enzyme,WRKY transcription factor NtEIG-D48, Avr9/Cf-9 rapidly elicited protein 74
		JA mediated signaling pathway	592	38	1.90E-03	TCONS_00016976, TCONS_00057641,TCONS_00052052, TCONS_00055412, TCONS_00081296	serine acetyltransferase 7, BOP3, WRKY DNA-binding protein, jasmonate ZIM-domain protein10, MAP kinase kinase
		L-phenylalanine catabolic process	50	7	1.79E-02	TCONS_00069919	phenylalanine ammonia-lyase 4
		Response to blue light	372	25	9.11E-03	TCONS_00051968	chloroplast FtsZ-like protein
		Response to far red light	324	24	4.19E-03	TCONS_00033254	chlorophyll a/b-binding protein
		Response to red light	285	26	1.38E-04	TCONS_00049275	alpha-expansin precursor
		SA mediated signaling pathway	732	42	5.65E-03	TCONS_00081296, TCONS_00055623, TCONS_00016976	MAP kinase kinase, LEB5, serine acetyltransferase 7
	MF	Phenylalanine ammonia-lyase activity	15	7	1.13E-05	TCONS_00069919	phenylalanine ammonia-lyase 4
		Serine-type endopeptidase inhibitor activity	34	10	4.64E-06	TCONS_00053724, TCONS_00009793	trypsin proteinase inhibitor precursor, cyclin-T1-3-like
		UDP-glucosyltransferase activity	581	39	4.20E-04	TCONS_00002352, TCONS_00039739, TCONS_00066391	glucosyltransferase, UDP-glucose, SA glucosyltransferase, flavonoid 3-O-glucosyltransferase
	CC	Chloroplast thylakoid membrane	1211	91	5.07E-14	TCONS_00032434, TCONS_00018745, TCONS_00051968	plastid transketolase, chloroplast pigment-binding protein CP24, chloroplast FtsZ-like protein
		Photosystem I reaction center	28	9	1.81E-06	TCONS_00002104, TCONS_00102030, TCONS_00067457	PSI-H precursor, photosystem I subunit XI, PSI-H precursor
		Photosystem II reaction center	6	4	1.91E-04	TCONS_00065835, TCONS_00027279	photosystem II protein T, photosystem II reaction center PSB28 protein

**Table 3:** Significant enriched KEGG pathways of the difference express unigene

Seed status	KEGG ID	Refence set	Query set	*P-value* (FDR)	KEGG pathway
Dormancy	ko00062	12	54	2.68E-04	Fatty acid elongation in mitochondria
	ko00910	15	105	2.13E-03	Nitrogen metabolism
	ko04330	14	119	2.30E-02	Notch signaling pathway
	ko04075	62	1029	4.07E-02	Plant hormone signal transduction
Germination	ko00195	28	154	3.18E-12	Photosynthesis
	ko00980	25	131	1.80E-11	Metabolism of xenobiotics by cytochrome P450
	ko00940	39	370	1.55E-09	Phenylpropanoid biosynthesis
	ko00480	32	270	3.96E-09	Glutathione metabolism
	ko00196	14	78	4.20E-06	Photosynthesis - antenna proteins
	ko00360	29	315	6.65E-06	Phenylalanine metabolism
	ko04626	39	547	4.11E-05	Plant-pathogen interaction
	ko00941	10	65	8.04E-04	Flavonoid biosynthesis
	ko04976	9	63	3.31E-03	Bile secretion
	ko00592	12	123	1.01E-02	alpha-Linolenic acid metabolism
	ko00945	8	60	1.06E-02	Stilbenoid, diarylheptanoid and gingerol biosynthesis
	ko00965	2	2	1.90E-02	Betalain biosynthesis
	ko00052	17	256	5.00E-02	Galactose metabolism

The analyses for GO and KEGG pathway enrichment of genes in up-regulated or down-regulated auxin-responsive seeds were performed and it was found that these unigenes also showed different expression trend in primary dormancy seeds (*p-value* < 0.05*,* 3962; patterns_III+IV+VI+VII_). There were significant enrichment of GO terms related to auxin mediated signaling pathway (flavonol biosynthetic process, gibberellin metabolic process), adenylyl-sulfate reductase activity, phosphoadenylyl-sulfate reductase (thioredoxin) activity, and glutamate synthase (NADH) activity (Table 2 and Additional file 6: Figure S6). KEGG pathway enrichment analysis shown the differential express unigenes were significant enriched in plant hormone signal transduction (Additional file 7: Figure S7) fatty acid elongation in mitochondria, and so on (Table 3).

### Auxin-responsive germinating seeds and conventional germinating seeds differ in their gene expression profiles

**Fig. 9:**
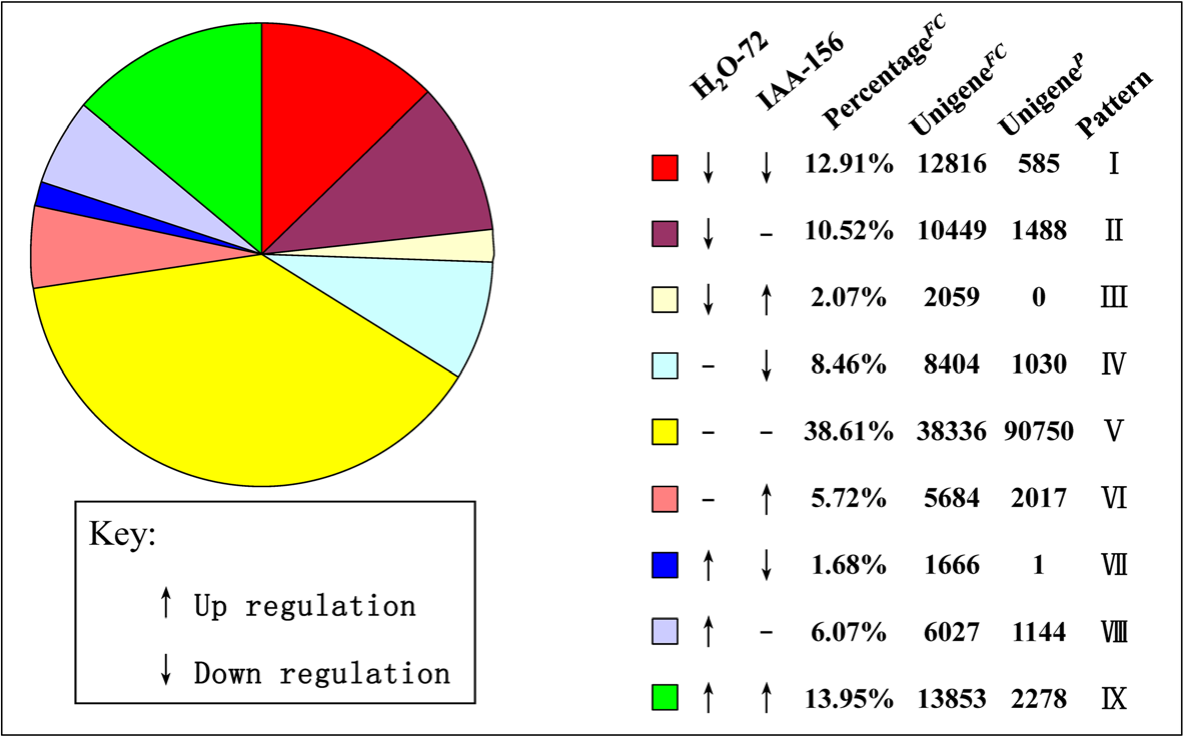
A pie chart distribution of unigenes whose expression is altered during seed germination. The pie chart was drawn with the percentage of fold change values. Unigenes whose expression level was significantly up-regulated or down-regulated by more than 2-fold or *p-value* < 0.05 during seed germination were grouped according to their expression behavior in the two germination states of H_2_O-72 seeds (normal germination) and IAA-156 seeds (auxin-imbibed germination) relative to IAA-72 seeds (secondary dormancy). Arrows facing up or down represent up-regulated or down-regulated genes, respectively

Using the same method as the dormancy experiment, a differential analysis between conventional germinated seeds (H_2_O-72) and auxin-responsive germinated seeds (IAA-156) relative to auxin-responsive dormancy seeds (IAA-72) was conducted. Firstly, at a significant level of *P < 0.05* and fold change ≥ 2, we identified 3526 up- and 2073 down-regulated unigenes between untreated seeds and H_2_O-imbibed seeds (Additional file 5: Figure S5C). At the same condition, we also identified 4458 up- and 1616 down-regulated unigenes between auxin-imbibed seeds and H_2_O-imbibed seeds (Additional file 5: Figure S5D). The transcriptome comparison analysis of two germinated states seeds of conventional germinated seeds (H_2_O-72) and auxin-responsive germinated seeds (IAA-156) in comparison with auxin-responsive dormancy seeds (IAA-72) was performed. The unigenes were further classified into 9 expression patterns (Fig. 9). Like the different expression analysis in the dormancy seeds above, only unigenes in III/IV/VI/VII patterns were analyzed to identify the genes with differential expression between sample IAA-156 and sample H_2_O-72. These genes could also be treated as the major causal factors that led to the difference in mechanism between auxin-responsive germinated seeds and conventional germinated seeds.

The GO and KEGG pathway enrichment of genes in the up-regulated or down-regulated auxin-responsive germinated seeds showed that these genes had different expression trend compared with conventional germinated seeds (*p-value* < 0.05*,* 3048; patterns_III+IV+VI+VII_). The enrichment of GO terms were associated with ABA, auxin, JA and SA-mediated signaling pathway, response to red, far red and blue light, serine-type endopeptidase inhibitor activity, phenylalanine ammonia-lyase activity, UDP-glucosyltransferase activity, and photosynthesis, etc. (Table 2 and Additional file 8: Figure S8). The KEGG enriched pathway were associated with glutathione metabolism, flavonoid biosynthesis (Additional file 9: Figure S9), and phenylpropanoid biosynthesis (Table 3).

**Table 4:** Fold changes of significant differential expressed unigenes revealed in auxin-responsive seed dormancy and germination regulation by RNA-seq and their verification by RT-PCR

Gene	Encoded protein	RNA-seq				RT-PCR			
		Untreated-0 vs. H_2_O-0	IAA-0 vs. H_2_O-0	H_2_O-72 vs. IAA-72	IAA-156 vs. IAA-72	Untreated-0 vs. H_2_O-0	IAA-0 vs. H_2_O-0	H_2_O-72 vs. IAA-72	IAA-156 vs. IAA-72
ABA and GA crosstalk									
TCONS_00064877	AHG3*(*Protein phosphatase 2C)	–	0.318	–	–	–	0.3407	–	–
TCONS_00038628	GA3ox1	0.072	–	6.848	–	0.0146	–	1.6385	–
TCONS_00082895	GA2ox2	–	–	–	13.934	–	–	–	5.5919
TCONS_00084330	CYP707A2	0.008	–	–		0.0273	–	–	–
ABA and auxin crosstalk									
TCONS_00033126	PIN4	–	–	10.190	5.358	–	–	3.9632	7.2267
Met pathway									
TCONS_00038414	Cysteine synthase	0.094	2.415	–	–	0.0386	2.8547	–	–
TCONS_00035466	AdoMet:Met S-methyltransferase	–	0.105	–	–	–	0.5236	–	–
TCONS_00000319	S-adenosylmethionine synthas	–	–	5.156	7.679	–	–	1.7983	4.4588
TCONS_00049344	Met synthase	–	–	4.748	5.275	–	–	1.9543	3.8106
TCONS_00052600	Serine acetyltransferase	–	–	–	19.437	–	–	–	–
Epigenetic regulators						–	–	–	–
TCONS_00072327	EFS (Histone H3 methyltransferase)	–	20.418	–	–	–	0.3253	–	–
TCONS_00057450	HUB1(C3HC4 RING finger)	–	6.917	–	–	–	0.7614	–	–

### Genetic expression difference of auxin-responsive seed dormancy and germination in the acquainted core metabolism pathways

The crosstalk of ABA and GA [12], and interaction of auxin and ABA [13] were the most important pathways controlling seed dormancy and germination. Other important dormancy regulators could be divided into four groups involved in seed maturation, hormonal action, dormancy and chromatin regulation [14]. To study the changes of these important genes and signal pathways, we utilized a blastx search against the NCBI NR database, the homolog unigenes of which in tobacco were identified. Express patterns of these unigenes’ were obtained from differential expression analyses, and the result showed that *EFS*, *HUB1* and *AHG3* in auxin-responsive seed dormancy were significantly up-regulated and down-regulated (Table 4).

Met metabolism is a housekeeping mechanism in all organisms, and also is central to seed germination [19]. As the methods above, unigenes in methionine metabolism was studied. Met synthase and serine acetyltransferase gene were significantly unregulated both in auxin-responsive germinated seeds and conventional germinated seeds (Table 4). Cysteine (Cys) synthase gene was up-regulated in auxin-responsive seed dormancy seeds and down-regulated in primary dormancy seeds (Table 4).

### Validation of auxin-responsive genes by quantitative real-time PCR

To validate RNA-seq results, quantitative real-time reverse transcription-PCR (qRT-PCR) was used to conduct the expression analysis of randomly assigned nineteen auxin-responsive genes in dormancy and germination. Table 4 and Additional file 10: Table S1 show the comparison between the qRT-PCR and RNA-seq analysis, showing that all the auxin-responsive genes tested and previously identified by RNA-seq were confirmed by qRT-PCR. The results showed a significant positive correlation between the two quantitative approaches of gene expression (Pearson correlation: *p* = 5.9E-3, *r* = 0.65; Spearman correlation: *p* = 5.2E-5, *ρ* = 0.84), indicating that the RNA-seq expression analysis performed is highly reliable.

## Discussion

Seed dormancy and germination are complex biological processes which are affected by both developmental and environmental factors. Auxin was recognized as a secondary dormancy hormone that controls seed dormancy and germination in *Arabidopsis* [13]. In this study, we found that tobacco seeds imbibed in a high concentration of exogenous auxin solution could promote seed dormancy and inhibit germination assayed by both restraining radicle protrusion and cotyledon greening. However, the microscopic scanning results implied that the seeds soaked in auxin solution were germinating as many follicles bubbles were found in cells whose vacuoles did not expand completely despite their treatment with ddH_2_O. The transcriptome PCA results also indicated that auxin-induced seeds are more similar to the seeds treated with ddH_2_O when compared to the primary dormant seeds. Seed dormancy has been defined as the incapacity of a viable seed to germinate under favorable conditions [1, 20]. So, this exogenous auxin-mediated seed dormancy can be referred to as an environmental model that prevents seed germination in an unfavorable condition.

Dormancy or germination depends on the balance between the resistance strength of the surrounding tissues and the growth force of the elongating radicle [14]. In this study, we found that exogenous auxin completely restrained radicle emergence, but not refrained endosperm rupture judged by the activity of β-1,3glucanase, a specific enzyme that was necessary for endosperm rupture during tobacco seed germination [21–23]. As recognized, radicle cell elongation was necessary for seed germination and was generally accepted to be sufficient for the completion of radicle protrusion, while cell division was not essential [24]. Thus, auxin level may also be the prerequisite for radicle development and emergence during seed germination. The seeds of triple *tir1afb2afb3* and the quadruple mutant *tir1afb1afb2afb3* [25] in *Arabidopsis* failed to develop a hypocotyl and root meristem. Therefore, high concentration of exogenous auxin in this study might also inhibit hypocotyl and root meristem development.

A recent study carried out by Liu et al. 2013 on *Arabidopsis*, revealed that auxin and ABA in seed dormancy are interdependent. Auxin acted upstream of the major regulator of seed dormancy by activating the ABA response, but the ABA biosynthesis was not stimulated [13]. This study also revealed, that seeds imbibed in superfluous exogenous auxin did not show increase in ABA level. GA and IAA levels significantly increased during the imbibition stage, and both of them progressively decreased in the subsequent germination stage. These findings suggest that seed in order to release form the dormant state, more GA synthesis is required to antagonize the application of the auxin. The GA pathway was shown to have been subjected to regulation by auxin, and the auxin promotes *Arabidopsis* root growth by modulating gibberellin response [26]. GAs, although required for the completion of germination, are not directly involved in many processes taking place during germination, which occurred at a stage coinciding with or very close to radicle emergence [19]. So, the exogenous auxin application might significantly promoted dormancy by activating the ABA response during seed dormancy, and also inhibited the radicle development and emergence by modulating gibberellin response during seed germination.

The induction of seed dormancy is controlled by a diverse group of regulators, which can be divided into four groups that may be involved in: i. seed maturation, ii. hormonal action, iii. dormancy and iv. chromatin regulation [14]. In present study, we found that a hormonal regulator *AHG3* was significantly down-regulated. AHG3/AtPP2CA functions as a negative regulator in the ABA-signaling pathway, suggesting that it plays a major role in ABA signaling in seed germination and early growth of *Arabidopsis* [27]. Moreover, expression levels of two epigenetic regulators *EFS* and *HUB1* were significantly up-regulated in this study. The *EFS* gene has been selected as a phase transition regulator during seed germination in a transcriptional network modelling study, and the mutant *efs* seeds also show a variety of seed phenotypes including precocious germination [28]. The *RDO4/HUB1* gene was initially identified on the basis of its reduced dormancy phenotype [29].

Met metabolism is metabolism central for seed germination [19]. During seed germination of *Arabidopsis* [30–32], rice [33, 34], and peas [35], Met synthase or adometsynthetase accumulation increased. In *Arabidopsis*, studies have shown that the accumulation level of Met synthase strongly increased prior to radicle emergence, but no further increase was observed during radicle emergence [30–32]. In this study, Met synthase and adometsynthetase gene were significantly up-regulated (refer to auxin-responsive germination). Serine acetyltransferase is a crucial enzyme in Cys synthesis metabolism that was significantly up-regulated in auxin-responsive germinated seeds. Cys is a precursor of Met biosynthesis [36] and constitutes a building block contributing to protein structure through the formation or reduction of disulfide bonds as catalyzed by Trxs. It is well documented that these enzymes affect a myriad of proteins during germination [37]. Cys is also the precursor of the major antioxidant molecule glutathione (GSH), which is involved in several processes playing a role in germination. The GSH-ascorbate cycle [38] or the formation of S-nitroso glutathione (GSNO), or a storage form of NO plays a pivotal role in seed physiology [39]. Above all, auxin-responsive germination refers to an enhanced met pathway in the transcriptome.

## Conclusions

Unlike controlling of seed dormancy by exogenous ABA in a microscale, this exogenous auxin-mediated seed dormancy is more likely to be an environmental model that prevents seed germination in an unfavorable condition. Compared to the cotyledon, the radicle was more sensitive to exogenous auxin stimulation. Radicles of seeds that imbibed in 1 g/L exogenous auxin solution could not develop normally and emerge. In response to exogenous auxin stimulation, seeds would stimulate more GA synthesis to antagonize the effect of auxin. As the auxin level decreased, seeds recovered from the dormancy status to a germination status. Principal component analysis revealed that the transcriptome of auxin-responsive dormancy seeds was more similar to that of imbibed seeds when compared to primary dormancy seeds. And the transcriptome of auxin-responsive germinated seeds was more similar to that of conventional germinated seeds when compared to auxin-responsive dormancy seeds. To obtain the signaling pathways induced by auxin, the unigenes of showed differential expression between untreated seeds and auxin-imbibed seeds relative to H_2_O-imbibed seeds were chosen, was used to do gene function analysis. Auxin-responsive dormancy was associated with flavonol biosynthetic process, gibberellin metabolic process, adenylyl-sulfate reductase activity, thioredoxin activity, glutamate synthase (NADH) activity and chromatin regulation. Auxin-responsive germination responded to ABA, auxin, JA and SA mediated signaling pathway (red, far red and blue light), glutathione and methionine metabolism, of which most importantly we found that the auxin-responsive secondary seed dormancy refers to epigenetic regulation and germination to enhance Met pathway. Our study, thus, uncovers a previously unrecognized transcriptional regulatory networks and physiological development processes of seed dormancy and germination with superfluous auxin signal activate.

## Methods

### Seed imbibition; germination and radicle traits measurement

Seeds of tobacco (*Nicotianatabacum L.*)Nanjiang3 were obtained from Guizhou Academy of Tobacco Science. Seeds were surface sterilized with 1 % CuSO_4_ solution for 30 min and then 0.5 % ZnSO_4_ solution for 15 min on a shaker and then washed three times with double distilled water for two minutes each. Sterile seeds were imbibed in a supplementation hormone solution of (0, 10, 100, 1000) mg/l IAA for 24 h in a 12 h light/12 h dark cycle at 25 °C ± 1 °C, with untreated seeds as control. The samples were washed three times with distilled water. Then, the seeds in each treatment were sown on the surface of paper bed in 90-mm-diameter plastic petri dishes and incubated in a 12 h light/12 h dark cycle at 25 °C ± 1 °C. Germination was defined as visible radicle emergence to seed length. After 156 h, ten radicles, randomly sampled from germinated seeds, were weighed on a 10^−3^ g balance, and radicle length and surface area measured with WinRHIZO [40].

### Extraction, purification and quantification of the phytohormones and β-1,3glucanase activity in seeds

The 0, 36, 72, 108 and, 144 h geminating seeds were collected for β-1,3glucanase activity quantification and 0, 72 and, 144 h geminating seeds for phytohormones ABA, GA_1+3_ and IAA quantification. The method for extraction, purification, and quantification of phytohormones was modified from the description of Wang [41]. ELISA kits used for estimation of the hormonal levels came from China Agricultural University (Beijing, China). The procedures for proteins extraction were modified from the Leubner-Metzger described [21]. ELISA kits used for the estimation of the β-1,3glucanase activity came from R&D Systems parent company (Minneapolis, America).

### Scanning the vacuole of imbibed seeds by electron microscope

Among the imbibed samples, 0 and 1000 mg/L IAA treated samples were used for subcellular structure observation. Approximately half of the seeds were fixed in FAA (Formalin-Acetic - Alcohol) buffer and exhausted with an aspirator pump. Subsequently, serial transverse sections from the paraffin-embedded tissue were sequentially stained with safranin and fast green. Finally, these sections were observed with a transmission electron microscope (JEOL 1230, JEOL Ltd, and Japan).

### RNA extraction and next generation sequencing

The total RNA from the tobacco seeds was extracted using total RNA purification kit (LC Science, TRK-1001) according to the manufacturer’s instructions. The integrity and quality of the total RNA were checked using NanoDrop 2000 Spectrophotometer (Thermo Scientific, USA) and formaldehyde agarose gel electrophoresis. RNA was only used when the Abs260 nm/Abs280 nm ratio was >1.8. Constructing database standards were as follows: RIN value ≥ 7.5, RNA content ≥ 15 μg and, concentration ≥ 300 ng/μl.

mRNA were enriched from 5ug qualified total RNA using Invitrogen Dynabeads mRNA Ditect kit, and then mRNA were fragmented on block at 95 °C for 2mins followed by the addition of stop solution to end the reaction. After purification by Qiagen kit, the RNA fragments were used to first strand cDNA synthesis by SMARTscript II reverse transcriptase. Afterward, SMARTeroligos and dNTPs were added to synthesize double cDNA. After gel purification of cDNA, purified products were used as template to generate sequencing library. We used qPCR to check library quality and calculate library concentration. Libraries were sequenced with Illumina HiSeq 2000 platform, each sample yielding 10Gb data from the final library fragments using V3 reagent. Base calling was performed by CASAVA 1.8 software (Illumina).

### Transcriptome assembly with reference genome and functional classification

The RNA-Seq reads generated by the Illumina Genome Analyzer were initially processed to remove the adapter sequences and low-quality bases at the 3′ end. After preprocessing the RNA-Seq data, the reads were mapped to the K326 genome using a spliced aligner called Tophat [42] that can be used to identify novel splicing events and generate novel transcripts. Tophat with default parameters which allow up to two mismatches and report up to 40 alignments for reads mapping at multiple positions was run. The sam files generated by Tophat were provided as input to the software Cufflinks [18], which assembled the alignments in the sam file into transcripts. Then, Cufflinks were run with parameters to construct a minimum set of transcripts that best describes the RNA-Seq reads. Subsequently all libraries were assembled by Cufflinks. Cuffmerge [18] was used to merge these assemblies to generate a unique transcript sets, we called them as unigenes. Later, these unigenes were used to estimate express abundance (FPKM) by Cufflinks.

For unigenes function annotation, homology search with blastx algorithm was performed. Firstly, each transcript with length ≥ 200 bp was searched against NCBI NR database (ftp://ftp.ncbi.nih.gov/blast/db) and KOG database (ftp://ftp.ncbi.nih.gov/pub/COG/KOG/kyva) using blastx. The best similar hit with an E-value < 1.0e-5 was chosen to generate transcript annotation information. Secondly, each transcript with length ≥ 200 bp was annotated with GO database (http://www.geneontology.org/) with Blast2GO [43] that was based on the blast algorithm to obtain the Biological Process (BP), Cellular Component (CC) and Molecular Function (MF) GO terms information of annotated transcripts. Finally, all transcripts were searched against KEGG database with KAAS tool [44], and multiple plant organisms were chosen to get the KEGG best homologous ortholog IDs with the default parameters.

### Gene differential expression and gene enrichment analyses

For unigenes expression analysis, preprocessed RNA-seq reads were mapped to unique transcripts with Bowtie2, and then the unigene reads counts were obtained by eXpress, which can correct multiple mapped reads. Then, differential express transcripts between two treatments without replicate were detected by R DESeq package [45], which normalized the library size based on library reads counts and detected the DE genes based on the negative binomial distribution. nbinomTest ()function was used for no-replicate analysis. Differential express transcripts *p-value* was corrected by Benjamini and Hochberg FDR correction.

Over-representation of GO terms of differential express unigenes were identified by BiNGO plugin [46] in Cytoscape software with a hypergeometric test after Benjamini and Hochberg FDR correction at a significance level of *p-value* < 0.05 based on our custom tobacco transcripts GO annotated datasets.

Over-representation of KEGG pathways of differential express unigenes were identified with GSEAKEGGHyperGParams() function in R GOstats package [47], which was based on the hypergeometric test. *P-values* were corrected by Benjamini and Hochberg FDR method, then chose a significance level of *p < 0.05*. The KEGG native diagrams were obtained using keggview.native() function in R Pathview package [48].

### Real-time quantitative PCR

Quantification was performed with a two-step reaction process: reverse transcription (RT) and PCR. Each RT reaction consisted of 0.5 μg RNA, 2 μl of PrimerScript Buffer, 0.5 μl of oligo dT, 0.5 μl of random 6mers and 0.5 μl of PrimerScript RT Enzyme Mix I (TaKaRa, Japan), in a total volume of 10 μl. Reactions were performed in a GeneAmp® PCR System 9700 (Applied Biosystems, USA) for 15 min at 37 °C, followed by heat inactivation of RT for 5 s at 85 °C. The 10 μl RT reaction mix was then diluted 10 times in nuclease-free water and held at −20 °C.

Real-time PCR was performed using LightCycler® 480 II Real-time PCR Instrument (Roche, Swiss) with 10 μl PCR reaction mixture that included 1 μl of cDNA, 5 μl of 2 × LightCycler® 480 SYBR Green I Master (Roche, Swiss), 0.2 μl of forward primer, 0.2 μl of reverse primer and 3.6 μl of nuclease-free water. Reactions were incubated in a 384-well optical plate (Roche, Swiss) at 95 °C for 10 min, followed by 40 cycles of 95 °C for 10 s, and 60 °C for 30 s. Each sample was run in triplicate for analysis. At the end of the PCR cycles, melting curve analysis was performed to validate the specific generation of the expected PCR product. The primer sequences were designed in the laboratory and synthesized by Generay Biotech (Generay, PRC) based on the mRNA sequences obtained from the NCBI database (Additional file 11: Table S2). The expression levels of mRNAs were normalized to L25 and were calculated using the 2-ΔΔCt method [49].

### Statistics

The results are expressed as means ± standard deviation (SD) calculated from at least three replications per treatment. ANOVA for radicle traits, β-1,3glucanase activity and plant hormone levels were performed using the Ducan’s test (*P* < 0.05 error level). Statistical analyses were performed using SPSS software Ver.16.0, and plotting using Origin software Ver.8.5.

### Availability of supporting data

RNA-seq read data has been deposited in the NCBI SRA database under accession number SRP068795.

## Additional files

Additional file 1: Figure S1. The distribution of the unigenes’ best blastx hit E-value in dormancy and germination of *Nicotiana tabacum* L. plants after RNA-seq analysis. (TIF 9107 kb) Additional file 2: Figure S2. The distribution of best hits species was shown in dormancy and germination of *Nicotiana tabacum* L. plants after RNA-seq analysis. (TIF 7945 kb) Additional file 3: Figure S3. KOG classification observed in dormancy and germination of *Nicotiana tabacum* L. plants after RNA-seq analysis. (TIF 27158 kb) Additional file 4: Figure S4. Distribution of auxin-responsive genes from whole *Nicotiana tabacum* L. seeds in several GO categories. Genes with putative functions were assigned to (A) molecular function, (B) biological process or (C) cellular component categories using GO annotations from the TAIR databa. (TIF 19948 kb) Additional file 5: Figure S5. Volcano plots of significant genes in dormancy and germination of *Nicotiana tabacum* L. plants after RNA-seq analysis. The x-axis represents the average value and the y-axis represents log 2 fold change. Several breakpoints of the Fc values are indicated on the Y-axis, where 0 indicates ‘no change’. Up-regulated and down-regulated genes are shown in red (*p <0.05*) and genes with a slight change in expression are shown in black (Additional file 5: Figure S5A, Untreated-0 vs H_2_O-0; Additional file 5: Figure S5B, IAA-0 vs H_2_O-0; Additional file 5: Figure S5C, H_2_O-72vs IAA-72; Additional file 5: Figure S5D, IAA-156 vs IAA-72.). (ZIP 1914 kb) Additional file 6: Figure S6. Example of GO term enriched in the gene differential expression analyses of auxin-responsive dormancy seed. Enriched GO terms were identified using BinGO, and the network was visualized with Cytoscape. Colors of the circles indicate the *p-value* (Hypergeometric test with Benjamini and Hochberg FDR correction) of enrichment. The size of circles represents the background gene counts of GO terms. The complete GO enrichment list were shown in Table 2. (TIF 583 kb) Additional file 7: Figure S7. Differential expressed unigenes enriched in plant hormone signal transduction KEGG pathway in auxin-responsive dormancy seed (FDR adjusted *p-value* = 4.07E-02). Enriched KEGG pathway was identified by hypergeometric test with a significance level of FDR corrected *p-value* < 0.05. Colors of the gene rectangles indicate the gene express patterns which were shown as the legend. The complete KEGG pathway enrichment list were shown in Table 3. (TIF 666 kb) Additional file 8: Figure S8. Example of GO term enriched in the gene differential expression analyses of auxin-responsive germination seed. Enriched GO terms were identified by BinGO plugin in Cytoscape software, as described in “Methods”. Colors of the circles indicate the *p-value* of enrichment. The size of circles represents the background gene counts of GO terms. The complete GO enrichment list were shown in Table 2. (TIF 745 kb) Additional file 9: Figure S9. Differential expressed unigenes enriched in flavonoid biosynthesis KEGG pathway in auxin-responsive germination seed (FDR adjusted *p-value* = 8.04E-04). Enriched KEGG pathway was identified by hypergeometric test with a significance level of FDR corrected *p-value* < 0.05. Colors of the gene rectangles indicate the gene express patterns which were shown as the legend. The complete KEGG pathway enrichment list were shown in Table 3. (TIF 600 kb) Additional file 10: Table S1. Validation of auxin-responsive genes by RT-PCR. (DOCX 22 kb) Additional file 11: Table S2. The primer sequences of genes used for RT-PCR validation. (DOCX 22 kb)
