# Supplementary figures and images for: Exogenous auxin regulates multi-metabolic network and embryo development, controlling seed secondary dormancy and germination in Nicotiana tabacum L
Source: BMC Plant Biol. 2016 Feb 9;16:41. doi: 10.1186/s12870-016-0724-5 (PMC4748683; doi:10.1186/s12870-016-0724-5)

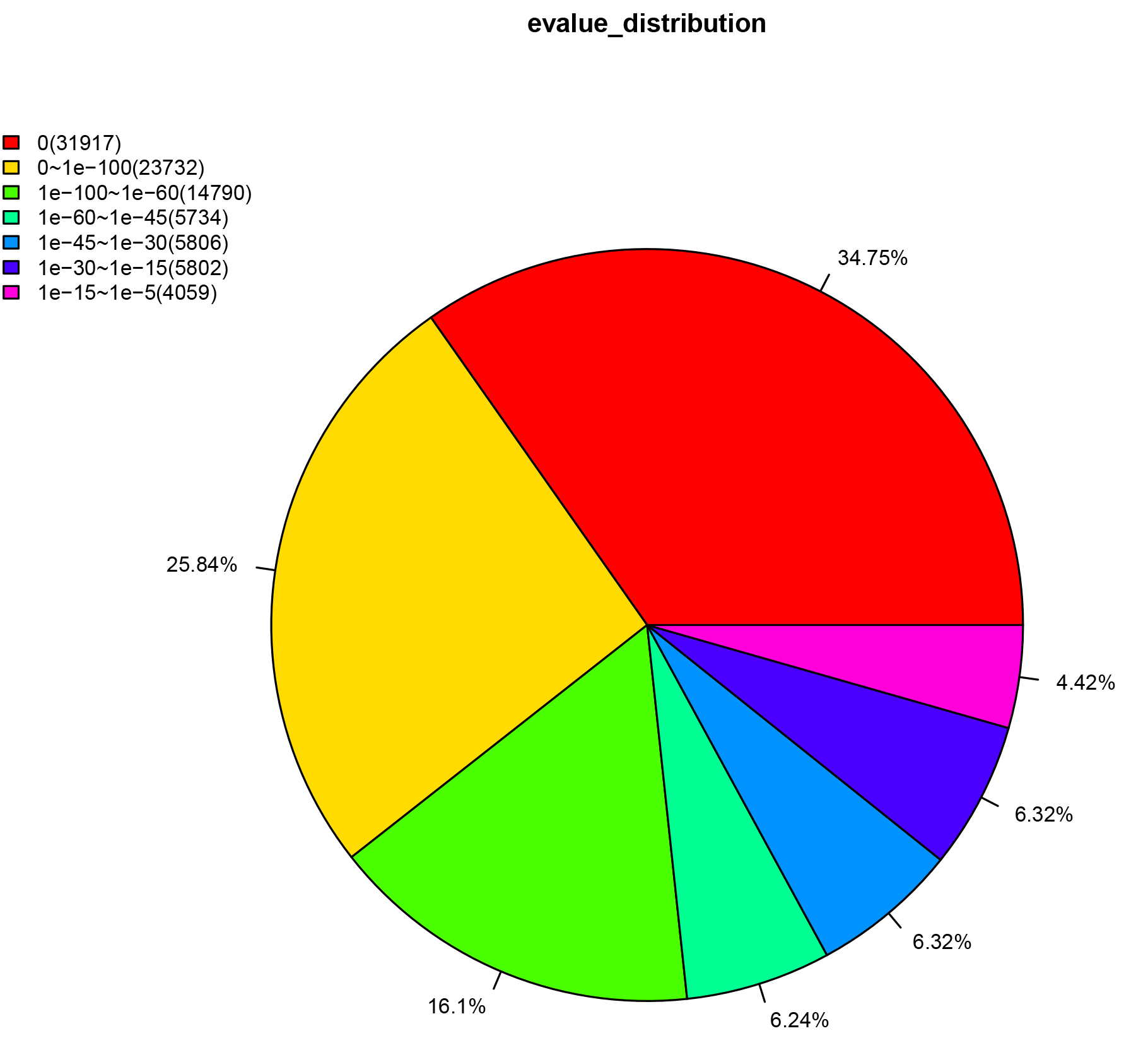

Supplement: Additional file 1: Figure S1. — The distribution of the unigenes’ best blastx hit E-value in dormancy and germination of Nicotiana tabacum L. plants after RNA-seq analysis. (TIF 9107 kb) [file 12870_2016_724_MOESM1_ESM.tif]

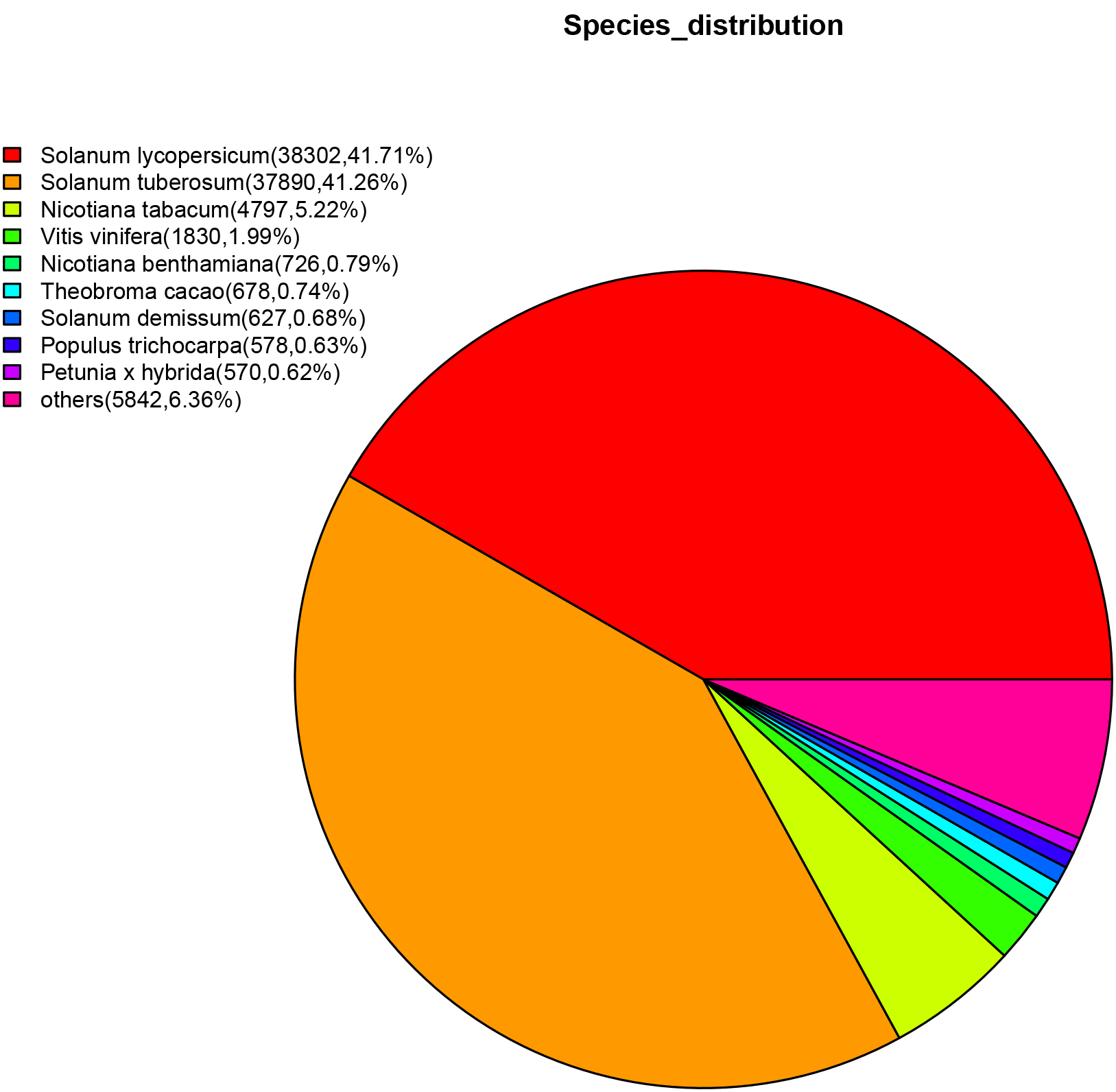

Supplement: Additional file 2: Figure S2. — The distribution of best hits species was shown in dormancy and germination of Nicotiana tabacum L. plants after RNA-seq analysis. (TIF 7945 kb) [file 12870_2016_724_MOESM2_ESM.tif]

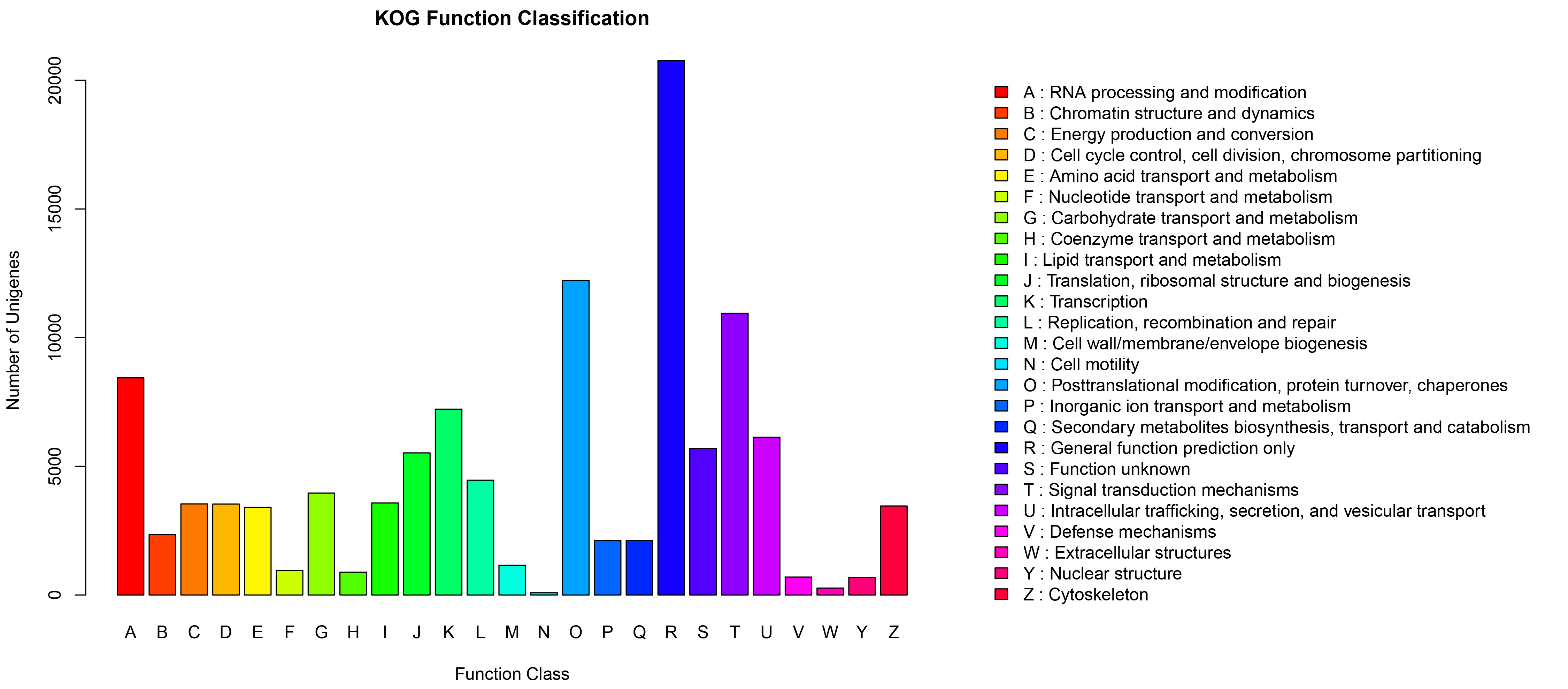

Supplement: Additional file 3: Figure S3. — KOG classification observed in dormancy and germination of Nicotiana tabacum L. plants after RNA-seq analysis. (TIF 27158 kb) [file 12870_2016_724_MOESM3_ESM.tif]

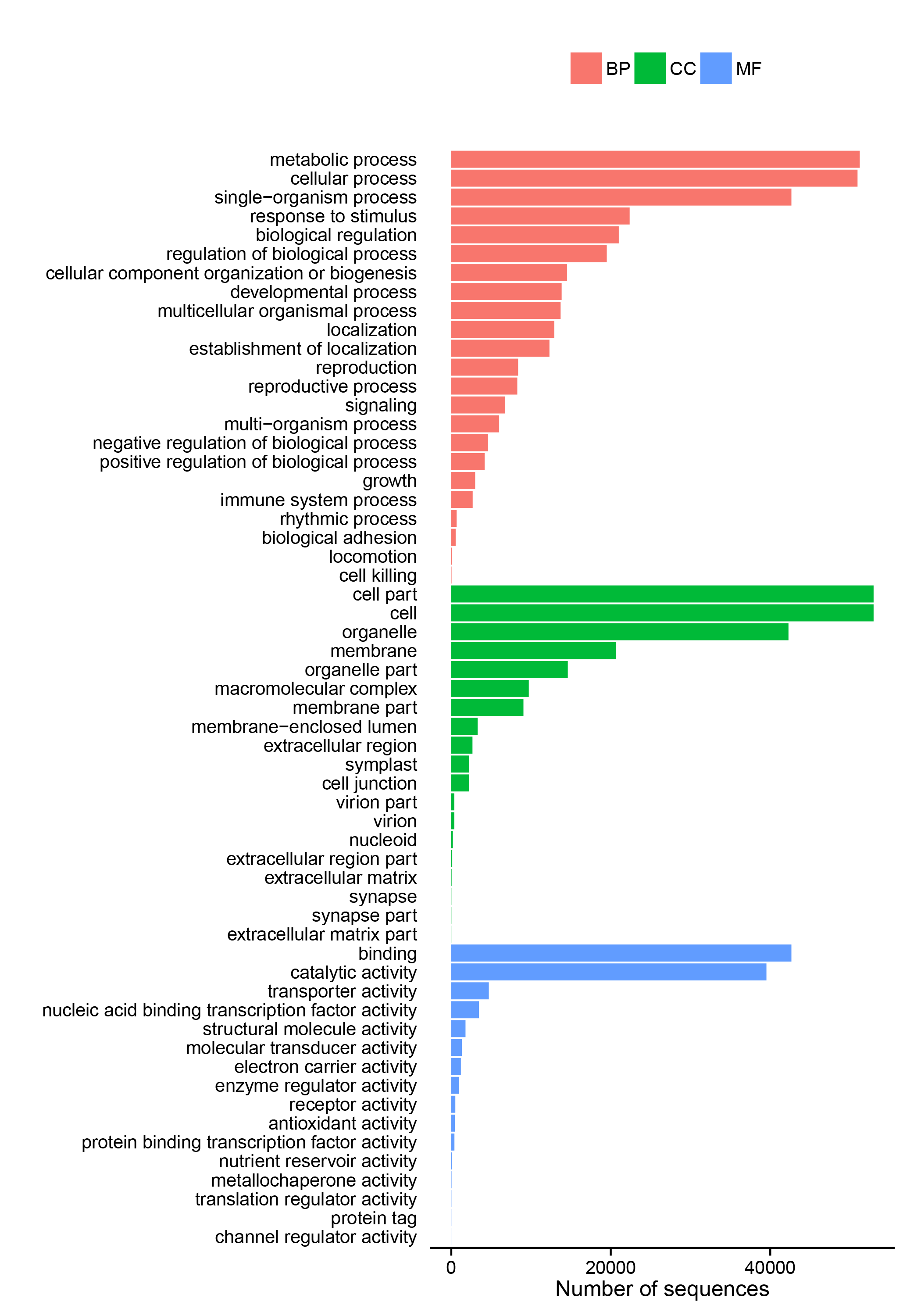

Supplement: Additional file 4: Figure S4. — Distribution of auxin-responsive genes from whole Nicotiana tabacum L. seeds in several GO categories. Genes with putative functions were assigned to (A) molecular function, (B) biological process or (C) cellular component categories using GO annotations from the TAIR databa. (TIF 19948 kb) [file 12870_2016_724_MOESM4_ESM.tif]

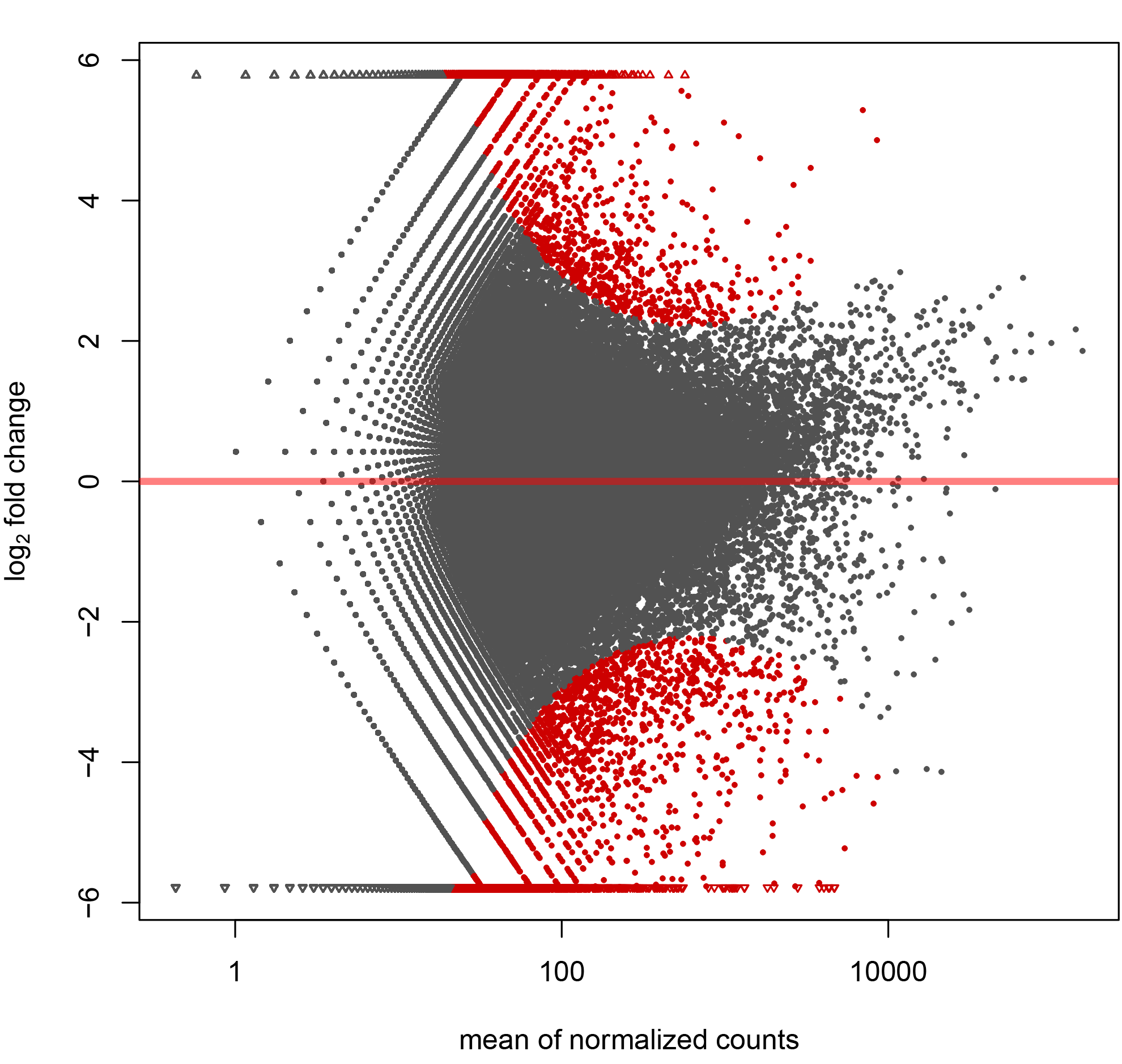

Supplement: Additional file 5: Figure S5. — Volcano plots of significant genes in dormancy and germination of Nicotiana tabacum L. plants after RNA-seq analysis. The x-axis represents the average value and the y-axis represents log 2 fold change. Several breakpoints of the Fc values are indicated on the Y-axis, where 0 indicates ‘no change’. Up-regulated and down-regulated genes are shown in red (p <0.05) and genes with a slight change in expression are shown in black (Additional file 5: Figure S5A, Untreated-0 vs H2O-0; Additional file 5: Figure S5B, IAA-0 vs H2O-0; Additional file 5: Figure S5C, H2O-72vs IAA-72; Additional file 5: Figure S5D, IAA-156 vs IAA-72.). (ZIP 1914 kb) [file 12870_2016_724_MOESM5_ESM.zip › FigS5A.tif]

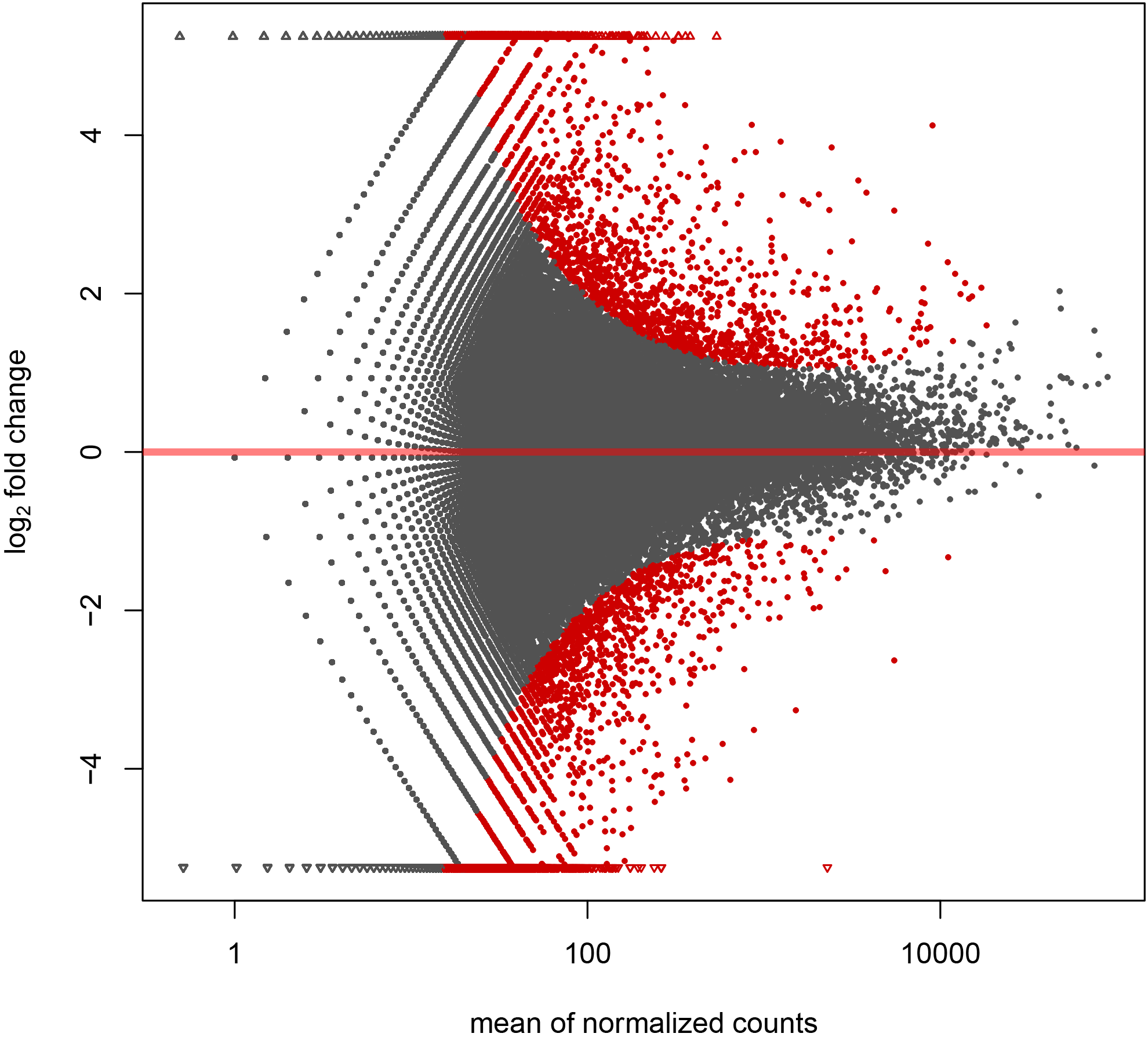

Supplement: Additional file 5: Figure S5. — Volcano plots of significant genes in dormancy and germination of Nicotiana tabacum L. plants after RNA-seq analysis. The x-axis represents the average value and the y-axis represents log 2 fold change. Several breakpoints of the Fc values are indicated on the Y-axis, where 0 indicates ‘no change’. Up-regulated and down-regulated genes are shown in red (p <0.05) and genes with a slight change in expression are shown in black (Additional file 5: Figure S5A, Untreated-0 vs H2O-0; Additional file 5: Figure S5B, IAA-0 vs H2O-0; Additional file 5: Figure S5C, H2O-72vs IAA-72; Additional file 5: Figure S5D, IAA-156 vs IAA-72.). (ZIP 1914 kb) [file 12870_2016_724_MOESM5_ESM.zip › FigS5B.tif]

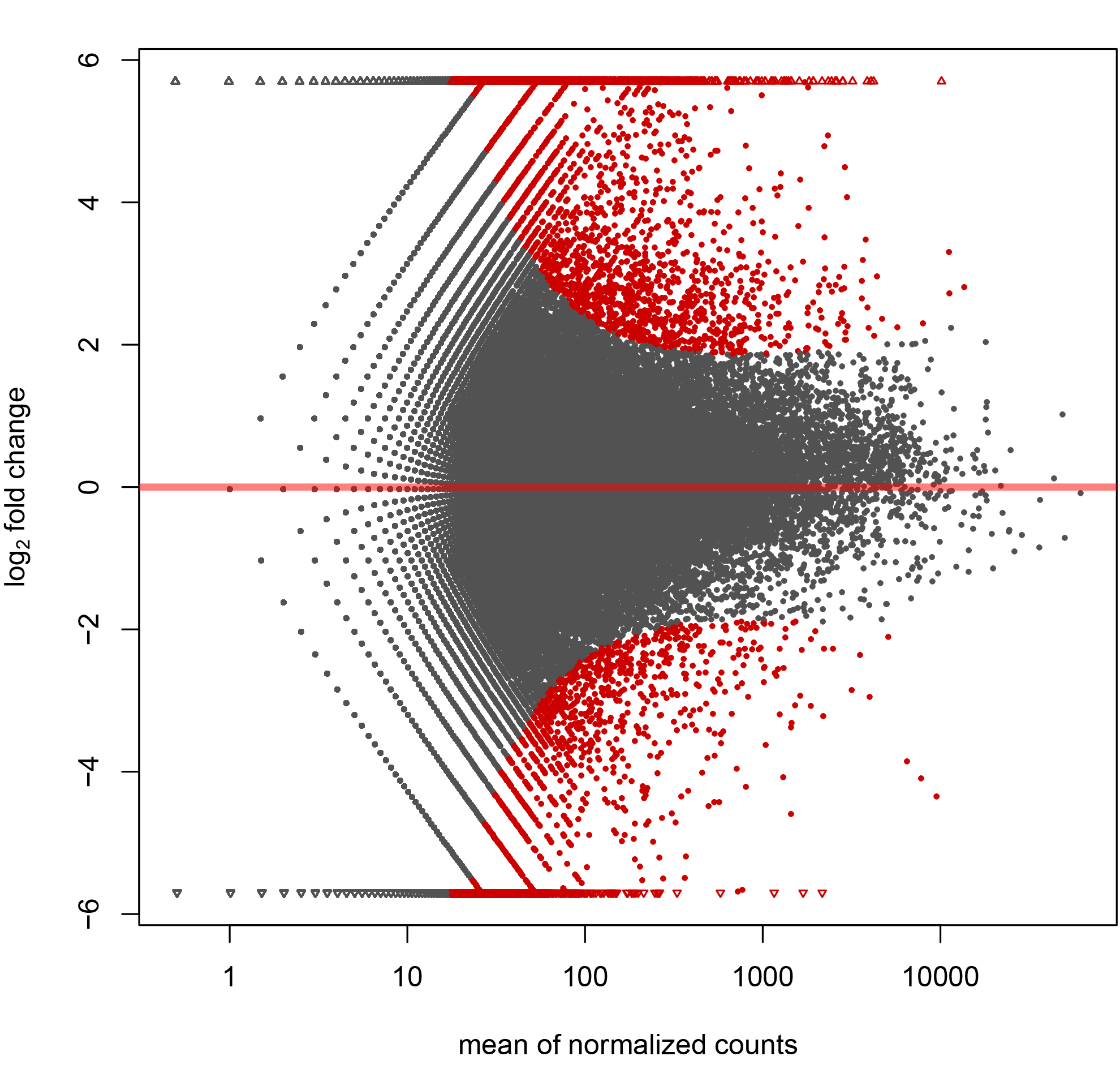

Supplement: Additional file 5: Figure S5. — Volcano plots of significant genes in dormancy and germination of Nicotiana tabacum L. plants after RNA-seq analysis. The x-axis represents the average value and the y-axis represents log 2 fold change. Several breakpoints of the Fc values are indicated on the Y-axis, where 0 indicates ‘no change’. Up-regulated and down-regulated genes are shown in red (p <0.05) and genes with a slight change in expression are shown in black (Additional file 5: Figure S5A, Untreated-0 vs H2O-0; Additional file 5: Figure S5B, IAA-0 vs H2O-0; Additional file 5: Figure S5C, H2O-72vs IAA-72; Additional file 5: Figure S5D, IAA-156 vs IAA-72.). (ZIP 1914 kb) [file 12870_2016_724_MOESM5_ESM.zip › FigS5C.tif]

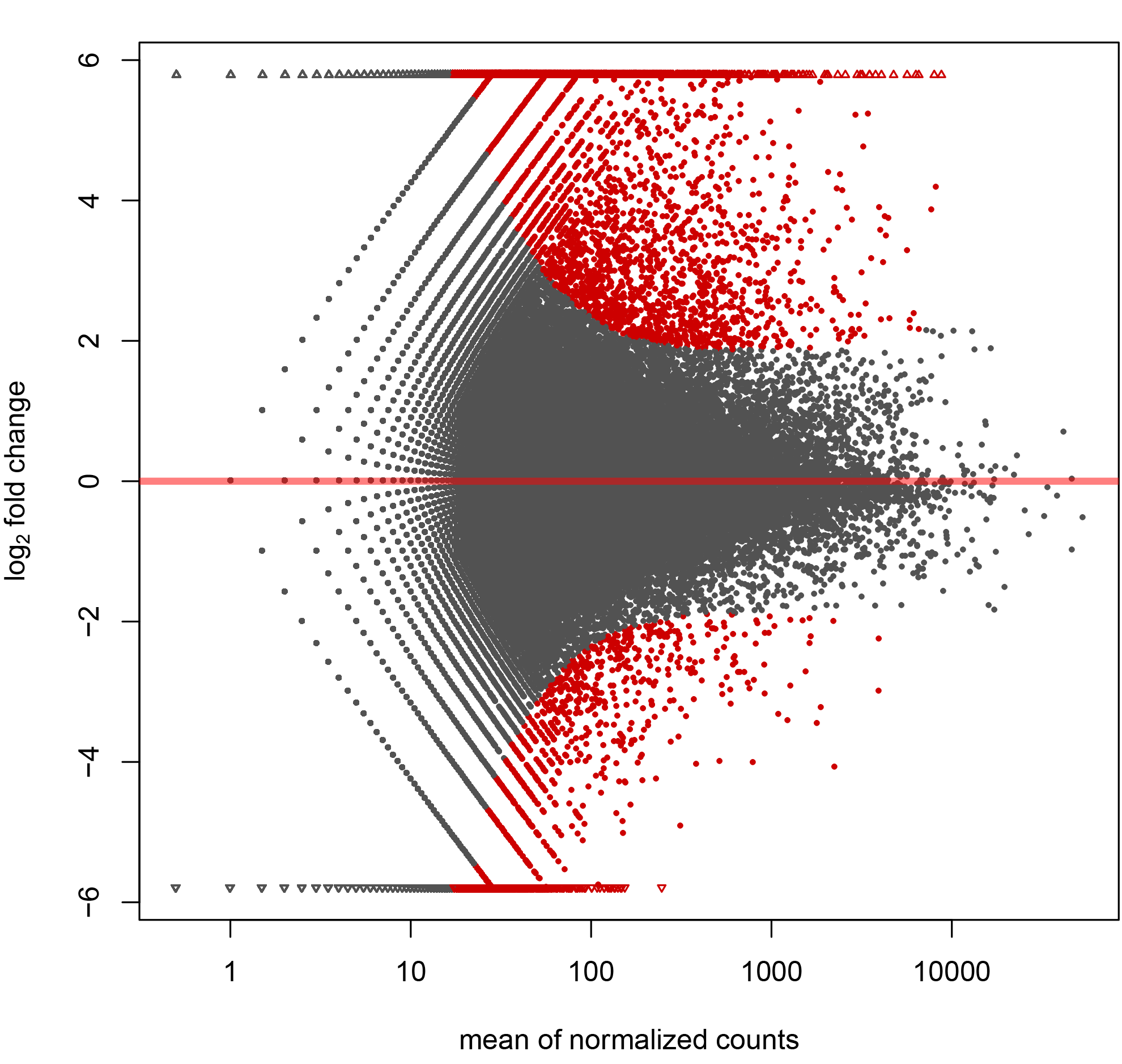

Supplement: Additional file 5: Figure S5. — Volcano plots of significant genes in dormancy and germination of Nicotiana tabacum L. plants after RNA-seq analysis. The x-axis represents the average value and the y-axis represents log 2 fold change. Several breakpoints of the Fc values are indicated on the Y-axis, where 0 indicates ‘no change’. Up-regulated and down-regulated genes are shown in red (p <0.05) and genes with a slight change in expression are shown in black (Additional file 5: Figure S5A, Untreated-0 vs H2O-0; Additional file 5: Figure S5B, IAA-0 vs H2O-0; Additional file 5: Figure S5C, H2O-72vs IAA-72; Additional file 5: Figure S5D, IAA-156 vs IAA-72.). (ZIP 1914 kb) [file 12870_2016_724_MOESM5_ESM.zip › FigS5D.tif]

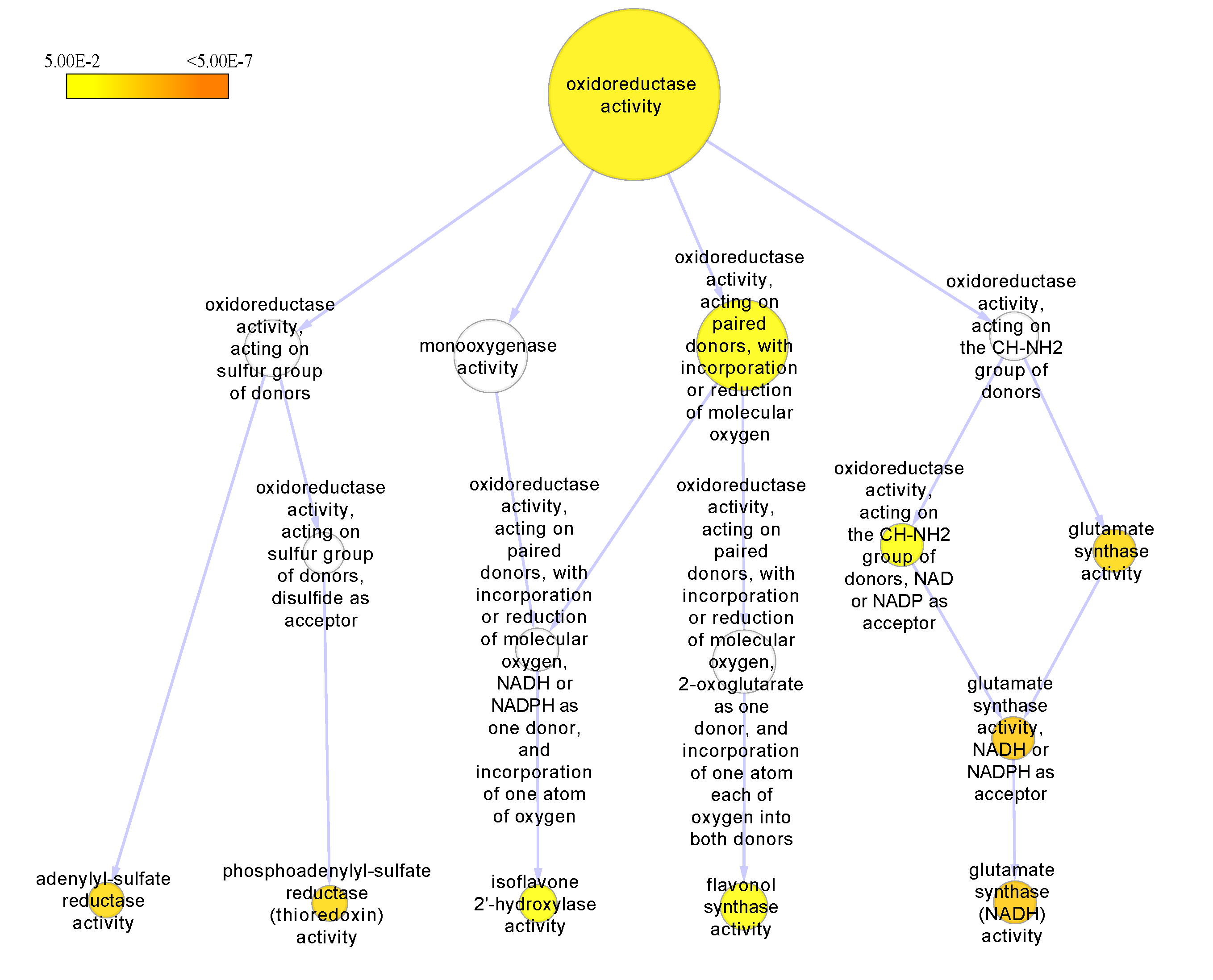

Supplement: Additional file 6: Figure S6. — Example of GO term enriched in the gene differential expression analyses of auxin-responsive dormancy seed. Enriched GO terms were identified using BinGO, and the network was visualized with Cytoscape. Colors of the circles indicate the p-value (Hypergeometric test with Benjamini and Hochberg FDR correction) of enrichment. The size of circles represents the background gene counts of GO terms. The complete GO enrichment list were shown in Table 2. (TIF 583 kb) [file 12870_2016_724_MOESM6_ESM.tif]

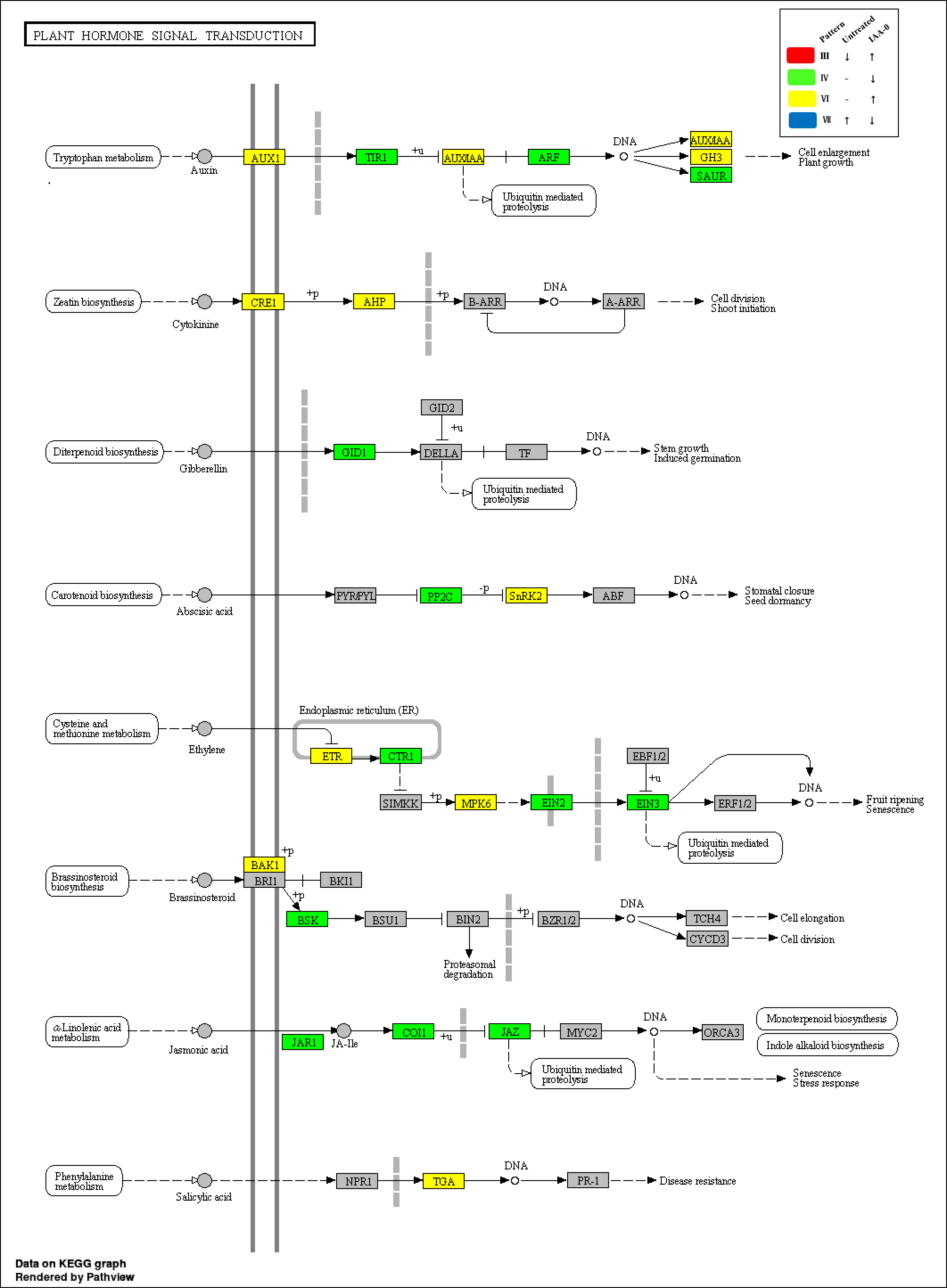

Supplement: Additional file 7: Figure S7. — Differential expressed unigenes enriched in plant hormone signal transduction KEGG pathway in auxin-responsive dormancy seed (FDR adjusted p-value = 4.07E-02). Enriched KEGG pathway was identified by hypergeometric test with a significance level of FDR corrected p-value < 0.05. Colors of the gene rectangles indicate the gene express patterns which were shown as the legend. The complete KEGG pathway enrichment list were shown in Table 3. (TIF 666 kb) [file 12870_2016_724_MOESM7_ESM.tif]

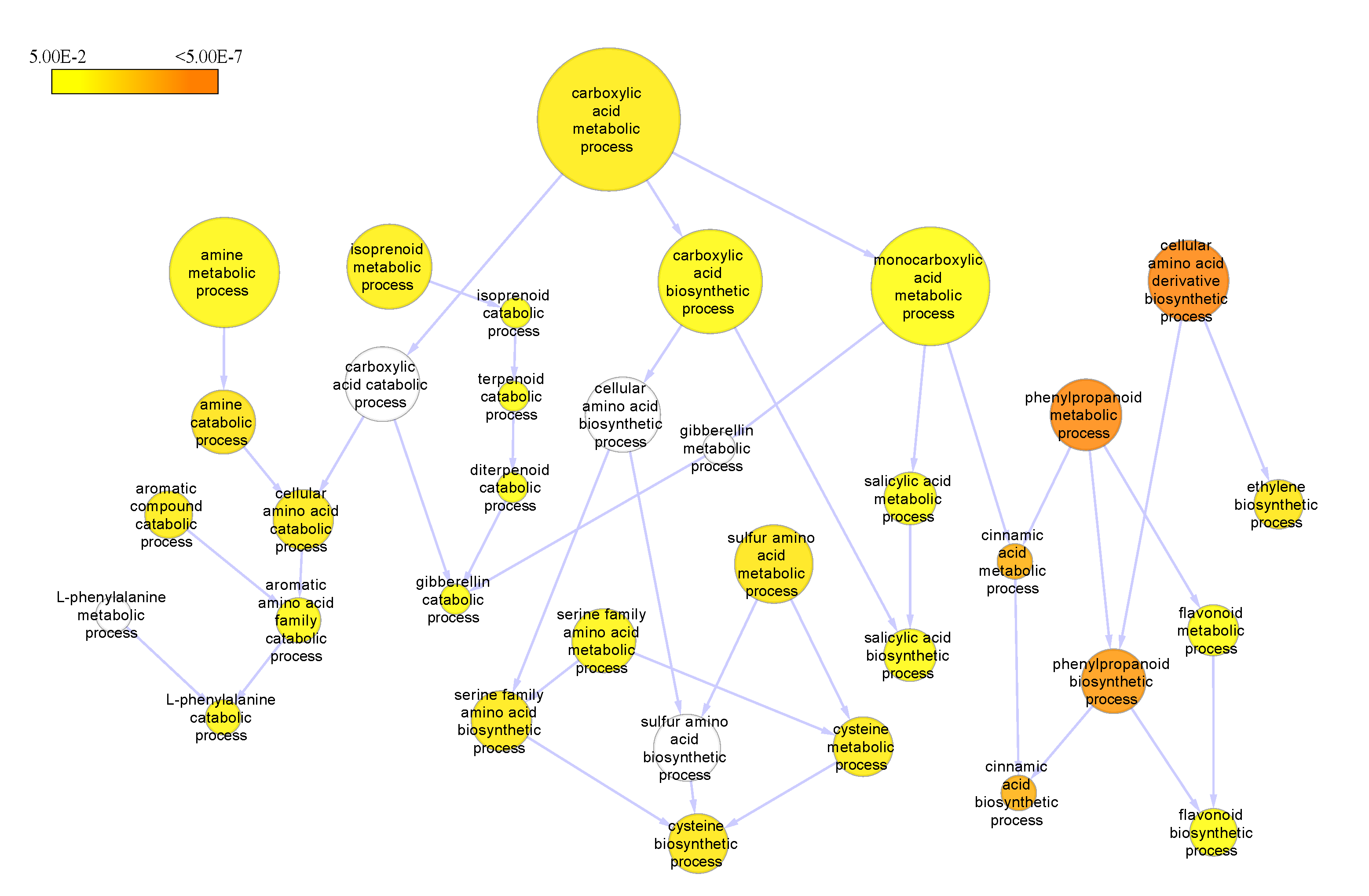

Supplement: Additional file 8: Figure S8. — Example of GO term enriched in the gene differential expression analyses of auxin-responsive germination seed. Enriched GO terms were identified by BinGO plugin in Cytoscape software, as described in “Methods”. Colors of the circles indicate the p-value of enrichment. The size of circles represents the background gene counts of GO terms. The complete GO enrichment list were shown in Table 2. (TIF 745 kb) [file 12870_2016_724_MOESM8_ESM.tif]

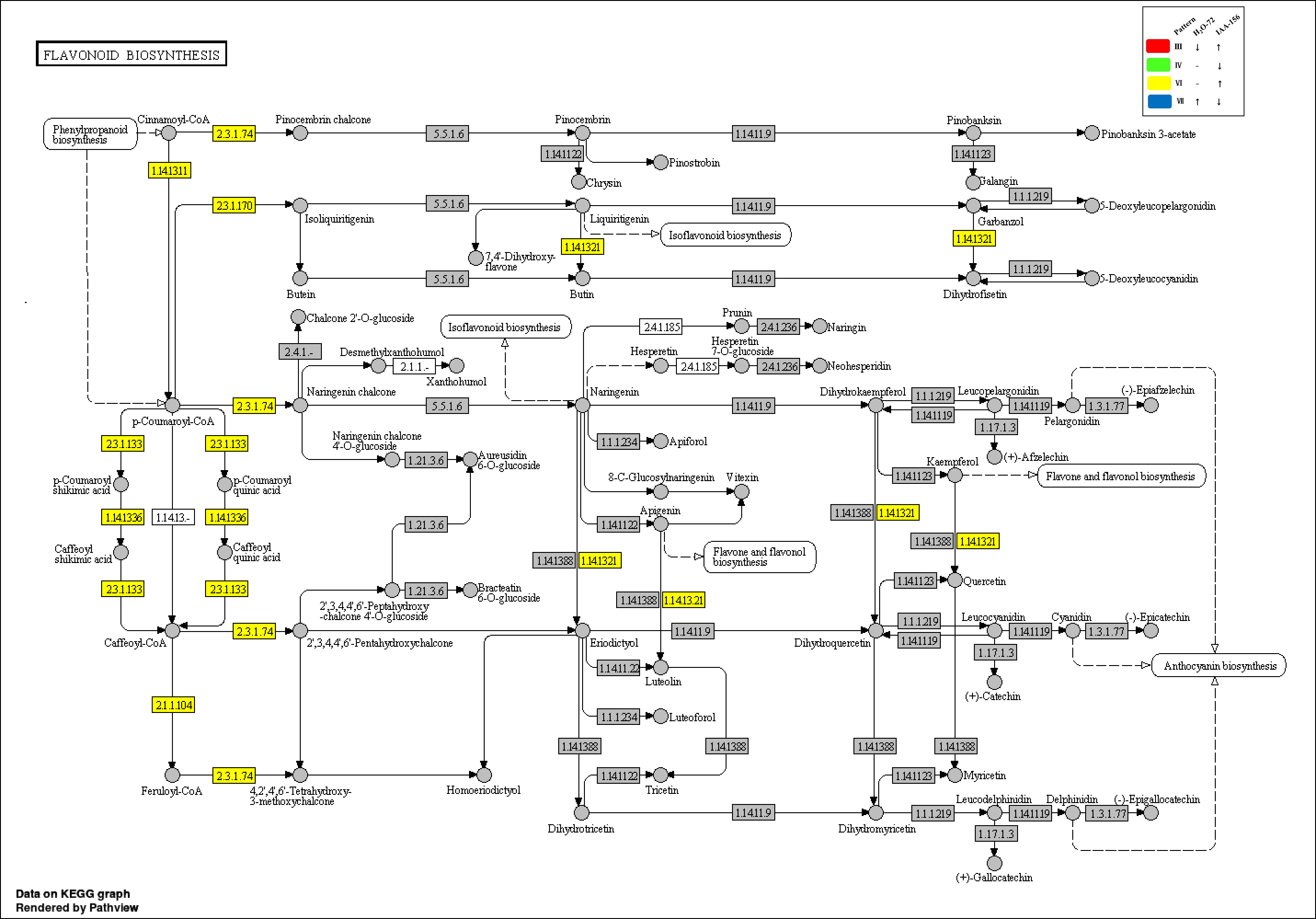

Supplement: Additional file 9: Figure S9. — Differential expressed unigenes enriched in flavonoid biosynthesis KEGG pathway in auxin-responsive germination seed (FDR adjusted p-value = 8.04E-04). Enriched KEGG pathway was identified by hypergeometric test with a significance level of FDR corrected p-value < 0.05. Colors of the gene rectangles indicate the gene express patterns which were shown as the legend. The complete KEGG pathway enrichment list were shown in Table 3. (TIF 600 kb) [file 12870_2016_724_MOESM9_ESM.tif]
