# Supplementary material for: Exogenous auxin regulates multi-metabolic network and embryo development, controlling seed secondary dormancy and germination in Nicotiana tabacum L
Source: BMC Plant Biol. 2016 Feb 9;16:41. doi: 10.1186/s12870-016-0724-5 (PMC4748683; doi:10.1186/s12870-016-0724-5)
Supplement: Additional file 10: Table S1. — Validation of auxin-responsive genes by RT-PCR. (DOCX 22 kb) [file 12870_2016_724_MOESM10_ESM.docx]

Table S1 Validation of auxin-responsive genes by RT-PCR.

| Sample | Gene | FPKM | ΔCt | 2^-ΔCt^ |
| --- | --- | --- | --- | --- |
| Untreated-0 | TCONS_00038628 | 0.8822567 | 9.45 | 0.001429771 |
| Untreated-0 | TCONS_00084330 | 0.04553829 | 10.4 | 0.000740096 |
| Untreated-0 | TCONS_00038414 | 3.191361 | 7.39 | 0.00596195 |
| IAA-0 | TCONS_00064877 | 14.1089 | 3.95 | 0.064704058 |
| IAA-0 | TCONS_00038414 | 85.70862 | 1.18 | 0.441351498 |
| IAA-0 | TCONS_00035466 | 0.2347152 | 4.88 | 0.033960465 |
| IAA-0 | TCONS_00072327 | 1.401096 | 9.31 | 0.001575472 |
| IAA-0 | TCONS_00057450 | 2.170447 | 4.49 | 0.044501569 |
| H_2_O-72 | TCONS_00038628 | 13.27623 | 5.65 | 0.01991501 |
| H_2_O-72 | TCONS_00033126 | 5.083854 | 6.19 | 0.013696964 |
| H_2_O-72 | TCONS_00000319 | 39.28504 | 3.98 | 0.063372468 |
| H_2_O-72 | TCONS_00049344 | 251.8193 | 2.71 | 0.152830035 |
| IAA-156 | TCONS_00082895 | 4.089661 | 4.65 | 0.03983002 |
| IAA-156 | TCONS_00033126 | 11.20643 | 5.33 | 0.024860515 |
| IAA-156 | TCONS_00000319 | 61.34934 | 2.67 | 0.157126672 |
| IAA-156 | TCONS_00049344 | 293.0663 | 1.75 | 0.297301779 |

Note: Pearson correlation: p=5.9E-3, r=0.65; Spearman correlation: p=5.2E-5, ρ=0.84
