# Supplementary material for: Exogenous auxin regulates multi-metabolic network and embryo development, controlling seed secondary dormancy and germination in Nicotiana tabacum L
Source: BMC Plant Biol. 2016 Feb 9;16:41. doi: 10.1186/s12870-016-0724-5 (PMC4748683; doi:10.1186/s12870-016-0724-5)
Supplement: Additional file 11: Table S2. — The primer sequences of genes used for RT-PCR validation. (DOCX 22 kb) [file 12870_2016_724_MOESM11_ESM.docx]

Table S2 The primer sequences of genes used for RT-PCR validation.

| **No.** | **Gene Symbol** | **Forward primer** | **Reverse primer** | **Product length(bp)** | **Ta(℃)** |
| --- | --- | --- | --- | --- | --- |
| 1 | TCONS_00069450 | CCAAAGATGAGACGTGTTACT | ACATCCTGAACCATTAGACC | 132 | 60 |
| 2 | TCONS_00090795 | GCAGGGATGTTGGAGATAG | TAGTCACCTTCAATCCGGG | 103 | 60 |
| 3 | TCONS_00038628 | AAACTAGCCGGAAGACTG | ATTTAGTTGTAGGGCCGAG | 114 | 60 |
| 4 | TCONS_00082895 | AAGATAGCACCTTTGGCA | TGCAACTTTCTCAAATAGGACC | 135 | 60 |
| 5 | TCONS_00084330 | TCCTAGTGTCCTACATGCTG | ACTCTTGTAGTCAAAGGCATC | 123 | 60 |
| 6 | TCONS_00024720 | ATTGATGATAAGCTGACCGT | TTTCTAGTCCTTTGATACAGCG | 156 | 60 |
| 7 | TCONS_00033126 | ATATGCAAGTGATTCTAGCCT | CGCAATCTTATTCCATTTCCG | 122 | 60 |
| 8 | TCONS_00038414 | CCCAAATACCTTCCTGGAAC | TAACAGCAATATAAGCACCACA | 115 | 60 |
| 9 | TCONS_00035466 | CATTCTGGCGTTTCAGTG | AGCATGGCTTCTCTGATATT | 122 | 60 |
| 10 | TCONS_00000319 | ATGAGACTGTCACAAACGA | CGATGGGTTAAGGTGGAATATG | 113 | 60 |
| 11 | TCONS_00049344 | GAGAACTCACGTTCCGAT | CTTGGAGAGTGGATGTCATAGA | 101 | 60 |
| 12 | TCONS_00072327 | AGCACATCAATGGAGACAC | GCATTAAACCCACTCAGACTAT | 115 | 60 |
| 13 | TCONS_00057450 | ATTGTTGAGACTCGTCACC | ACGAAAGGCCCAGATATT | 112 | 60 |
| 14 | TCONS_00064877 | GTCCTGAGGTTGTTTCTGG | CGAAGGGTGAACTGCTAC | 104 | 60 |
| 15 | TCONS_00046918 | CCCAGTACCTCAAATTAGTGT | CTCTTCCAACCACCAACG | 103 | 60 |
| 16 | TCONS_00057561 | AACAGGAAGAGACTTAGGCA | TGACCTCGATTCCAAAGAAATG | 111 | 60 |
| 17 | TCONS_00064315 | GCTTGTCGAATTGGTCTCTAA | GGAAATTGACTGTCAAGTGC | 108 | 60 |
| 18 | TCONS_00074927 | GGTGATGATAGCTCTTTACCAG | TCATGCAACATTAGCCGT | 119 | 60 |
| 19 | L25 | CCCCTCACCACAGAGTCTGC | AAGGGTGTTGTTGTCCTCAATCTT | 51 | 60 |
